# Structural Insights into the Regulation Mechanism of Small GTPases by GEFs

**DOI:** 10.3390/molecules24183308

**Published:** 2019-09-11

**Authors:** Sachiko Toma-Fukai, Toshiyuki Shimizu

**Affiliations:** 1 Graduate School of Science and Technology, Nara Institute of Science and Technology, 8916-5 Takayama-cho, Ikoma, Nara 630-0192, Japan; 2 Graduate School of Pharmaceutical Sciences, the University of Tokyo, 7-3-1 Hongo, Bunkyo-ku, Tokyo 113-0033, Japan

**Keywords:** small GTPases, GEF, crystal structure, regulation mechanism, local protein unfolding

## Abstract

Small GTPases are key regulators of cellular events, and their dysfunction causes many types of cancer. They serve as molecular switches by cycling between inactive guanosine diphosphate (GDP)-bound and active guanosine triphosphate (GTP)-bound states. GTPases are deactivated by GTPase-activating proteins (GAPs) and are activated by guanine-nucleotide exchange factors (GEFs). The intrinsic GTP hydrolysis activity of small GTPases is generally low and is accelerated by GAPs. GEFs promote GDP dissociation from small GTPases to allow for GTP binding, which results in a conformational change of two highly flexible segments, called switch I and switch II, that enables binding of the gamma phosphate and allows small GTPases to interact with downstream effectors. For several decades, crystal structures of many GEFs and GAPs have been reported and have shown tremendous structural diversity. In this review, we focus on the latest structural studies of GEFs. Detailed pictures of the variety of GEF mechanisms at atomic resolution can provide insights into new approaches for drug discovery.

## 1. Small GTPases and Their Regulators

### 1.1. An Overview

Guanine nucleotide binding proteins (G-proteins) regulate many cellular processes. A total of 37,767 proteins in 1383 genomes contain guanine nucleotide-binding domains (G domains) [1], including small G-proteins and heterotrimeric G-proteins. Small G-proteins are single-domain proteins that regulate aspects of cellular physiology including cell signaling, cell shape, motility, polarity, and vesicular transport. The larger heterotrimeric G-proteins consist of α, β, and γ-subunits, in which the α subunit has a conserved G domain and interacts with G-protein-coupled receptors (GPCRs) in order to mediate transmembrane signaling. Both small G-proteins and heterotrimeric G-proteins have critical roles in cellular processes; therefore, dysfunction of these proteins can cause severe disease. Small GTPases work throughout the cell, including at membranes, in the cytosol, and in the nucleus, and thus participate in many diverse cellular events. An understanding of small GTPase-activating mechanisms is therefore an important issue for both basic biology and drug discovery.

### 1.2. The GTP/GDP Cycle of GTPases by Their Regulators and PostTranslational Modifications

**Figure 1:**
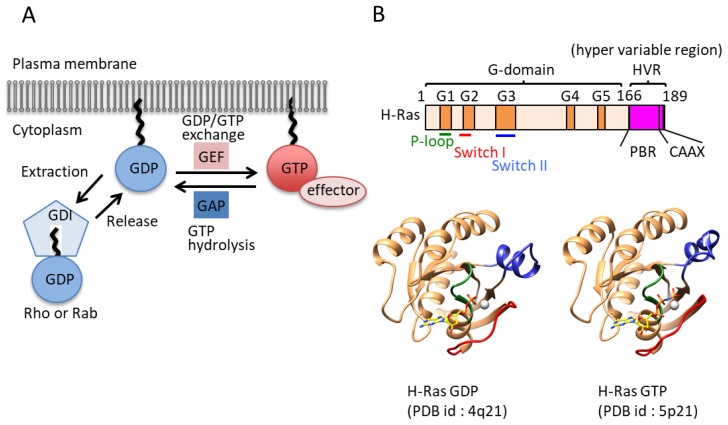
Regulation mechanism of small GTPases: (**A**) Schematic diagram of the small GTPase switching mechanism. Guanine-nucleotide exchange factors (GEFs) and GTPase-activating proteins (GAPs) enhance the exchange reaction. The guanine dissociation inhibitor (GDI) and effector are also shown. Guanosine triphosphate (GTP)-bound and guanosine diphosphate (GDP)-bound GTPases (lipidated forms) are shown as red and blue circles, respectively. GAP and GEF are shown as red and blue boxes, respectively. Several GTPase families combine their GDP/GTP switch with alternations in cytosolic/membrane localization in a process regulated by GDIs or GDI-like proteins. (**B**) General structural information of small GTPases. Upper: The domain architecture of H-Ras. G boxes of the G domain are highlighted with orange boxes. The hyper variable region, including a polybasic region and a CAAX motif, is highlighted with pink boxes. The P-loop, switch I, and switch II are shown as bars colored green, red, and blue, respectively. Lower: Crystal structures of GDP-bound and GTP-bound H-Ras. The P-loop, switch I, and switch II are colored green, red, and blue, respectively.

The first reported small GTPase was the Ras oncogene about three decades ago [2]. Small GTPases are typically 20–30 kDa in size and can work as molecular switches by alternating between a GTP-bound form and a GDP-bound form. Generally, the GTP-bound form is considered an active state and the GDP-bound form is considered an inactive form. Since the GTPase activity of G-proteins is intrinsically low, regulatory proteins are required for transitioning between active and inactive states. Guanine-nucleotide-exchange factors (GEFs) facilitate GDP dissociation while GTPase-activating proteins (GAPs) stimulate GTP hydrolysis (Figure 1A). GTP-bound small GTPases can bind effector proteins and can cause induction of a signaling response. This switching mechanism is cooperatively achieved by small GTPases, GEF, and GAP (Figure 1A) [3,4,5].

Another key regulator is guanine dissociation inhibitor (GDI). Some GTPases, including the Ras, Rho, and Rab proteins, are prenylated at their C-termini, and their C-terminal regions are known as hypervariable regions (HVRs), which contain a polybasic region (PBR) and four C-terminal residues called the C*aa*X motif, in which C represents a cysteine residue, *a* represents an aliphatic amino acid, and X represents any amino acid (Figure 1B). There are two prenylation enzymes: farnesyltransferase (FTase) and geranylgeranyltransferase (GGTase) [6,7]. Other motifs, such as CC, CCX, and CCXX, are modified by geranylgeranyltransferase II [8]. These lipid modifications occur in the cytosol [9], which allows for the trafficking of small GTPases to membranes, although this posttranslational modification is applied to a majority of the small GTPase superfamily. The existence of a GTPase with a specific modification in a certain location on a membrane is critical for normal biological activity. GDI can extract small GTPases from the membrane by binding prenylated inactive (GDP-bound) small GTPases. GDI forms a complex with its target GTPase and confines it to the cytosol (Figure 1A). Compared to GAPs and GEFs, a small number of GDIs have been identified, including RhoGDI (3 isoforms), RabGDI (3 isoforms), and PDEδ (phosphodiesterase-δ).

### 1.3. Small GTPase Families (Ras Superfamily)

Over the course of thirty years, a large number of small GTPases have been identified, including 114,619 proteins (564 proteins in humans) that have been divided into 8 subfamilies (Arf, Ran, Rab, Rho, Ras, Sar1, mitochondrial Rho, and mitochondrial Roc) using the program InterPRo [10]. ADP-ribosylation factor (Arf) proteins are involved in protein trafficking and modulate vesicle budding and uncoating within the Golgi apparatus. Ras-related nuclear (Ran) proteins are involved in nucleocytoplasmic transport. Ras related in brain (Rab) proteins are involved in vesicular trafficking. Ras homologous (Rho) proteins control cytoskeleton reorganization. Ras proteins regulate cell signaling. Secretion-associated and Ras-related (Sar1) proteins are components of coat protein complex II (COPII), which promotes the formation of transport vesicles from the endoplasmic reticulum (ER). Mitochondrial Rho (Miro) domains have been found in mitochondrial proteins and are involved in mitochondrial trafficking. Additionally, Ras of complex protein (Roc) domains are always associated with the C-terminal Rock (COR) domain.

### 1.4. The G Domain and the Molecular Switch Function

Amino acid sequence comparisons of small GTPases from various species indicate that they have 30–55% sequence similarity [11]. Small GTPases are composed of a single domain of which the structure is highly conserved from yeast to mammals. There are 614 small GTPase structures, including some in complexes with regulatory proteins that have been deposited into the PDB (Protein Data Bank) as of 2016 (listed in InterPRo). The G domain consists of a six-stranded β-sheet and 5 α-helices and has conserved sequence motifs called the G1, G2, G3, G4, and G5 motifs (G boxes), which are responsible for binding guanine nucleotide. The G1 motif (GxxxxGK[S/T], where x is any amino acid) is also called the P-loop and is found in many nucleotide binding proteins, where it recognizes the β-phosphate and a Mg^2+^ ion of target nucleotides (Figure 1B) [12]. The G2 motif (Thr) makes contacts with the γ-phosphate and the Mg^2+^ ion [3]. The G3 motif (DxxGQ) harbors the Gln residue responsible for GTP hydrolysis [13]. The G4 motif (NKxD) and G5 motif (TSAK) make specific contacts with the guanine base to distinguish guanine from other nucleotides (Figure 1B) [3,14].

To work as molecular switches, small GTPases have to exhibit structural differences between inactive and active states. Thirty years ago, the structures of both states were first unveiled, providing structural insight into the switching mechanisms [15,16]. Today, the following exchange mechanism is widely accepted. Upon exchange of the nucleotide, major conformational changes take place within two regions termed switch I and switch II. Switch I is located between α1 and β2, and switch II is located between β3 and the beginning of α2. The G2 motif, which is involved in interactions with the γ-phosphate and Mg^2+^ ion of targets, is in the switch I region (Figure 1B). The G3 motif involved in hydrolysis exists in switch II. Both regions show ordered conformations in the GTP-bound state (active form) and show a variety of conformations in the GDP-bound state (inactive form) [3].

## 2. GEF Structures and Mechanisms of Small GTPase Activation

### 2.1. An Overview

**Figure 2:**
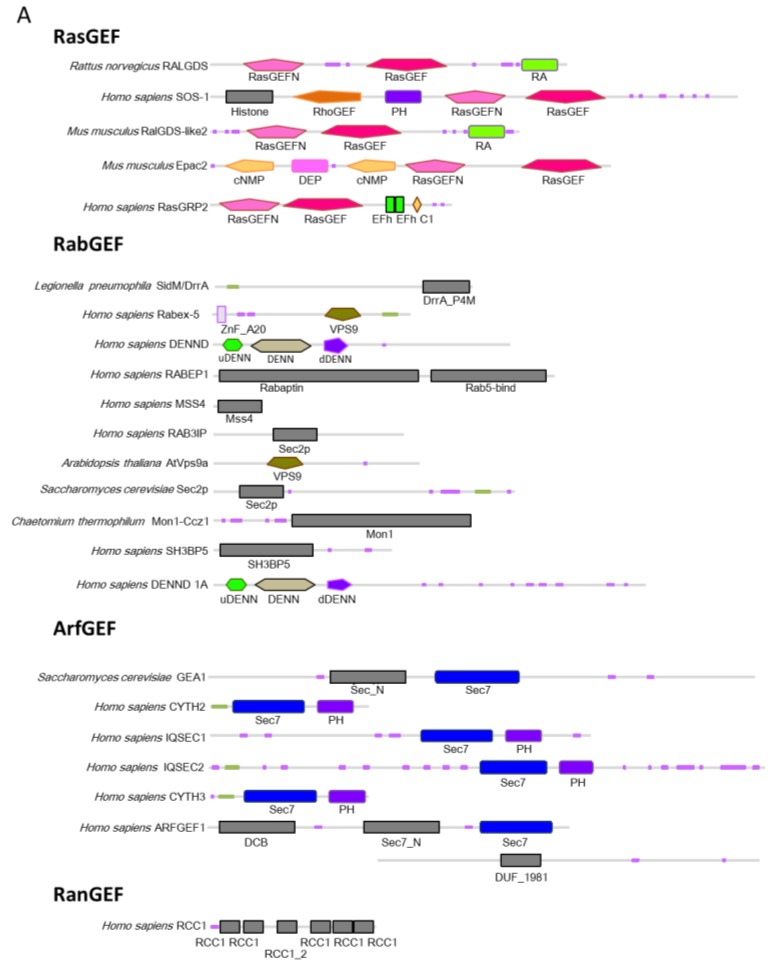

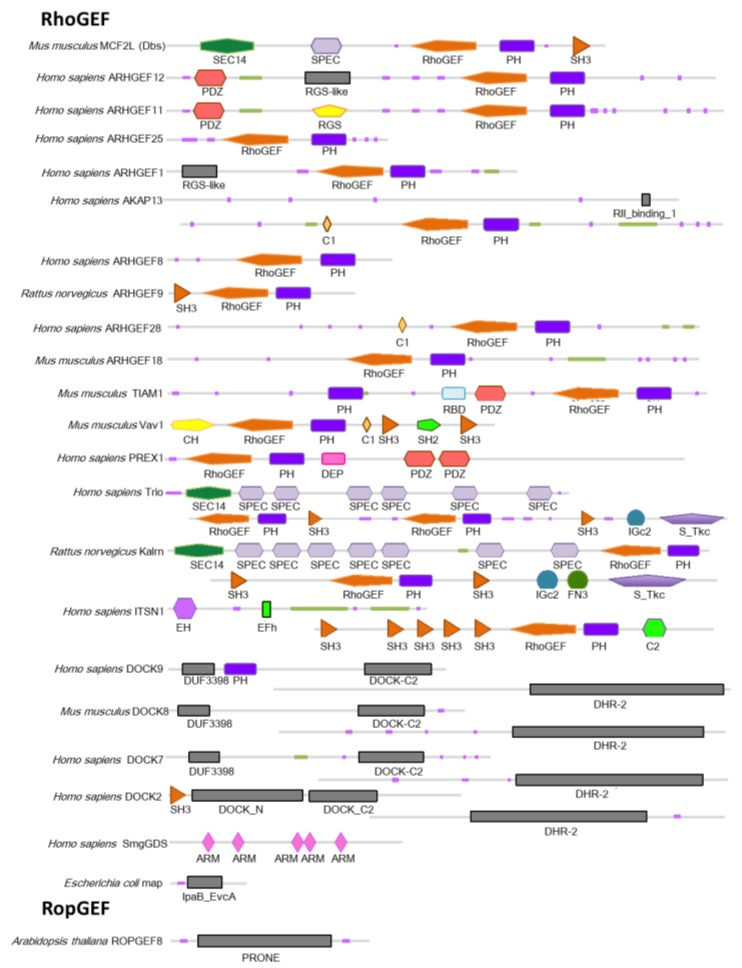

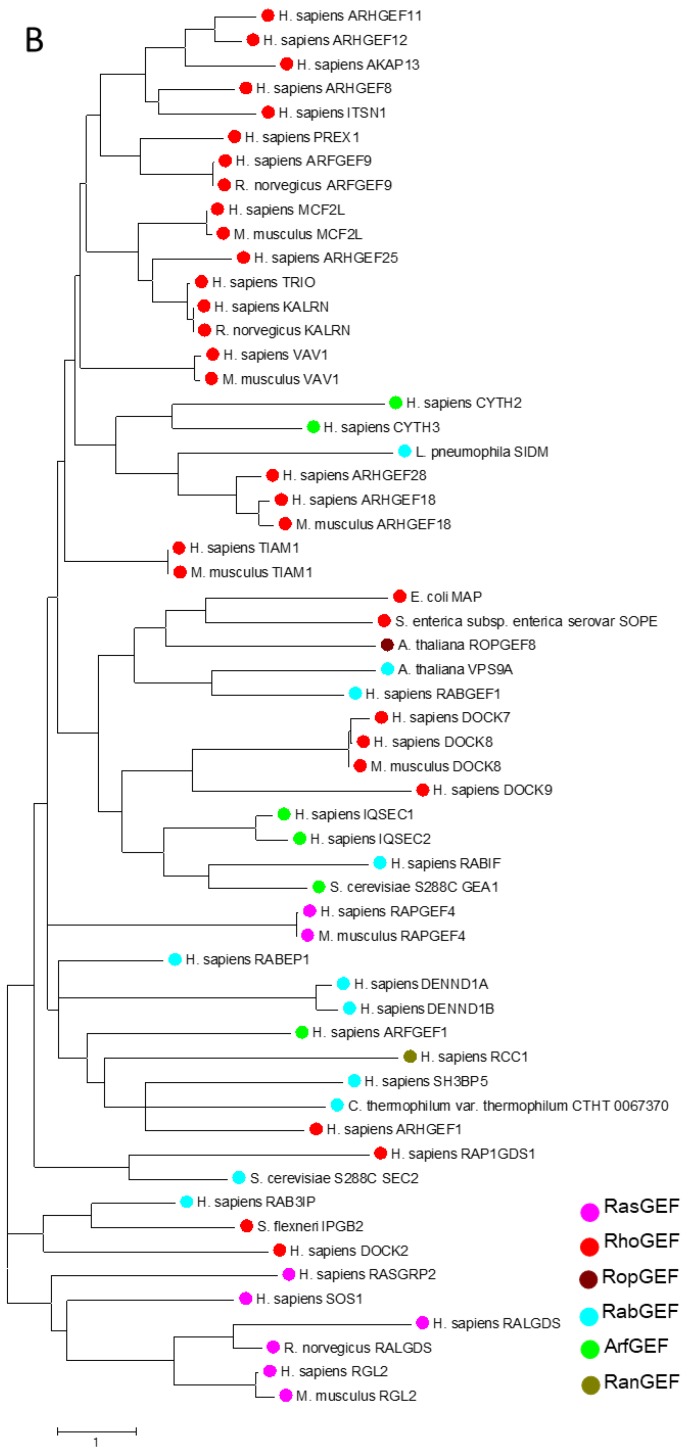
Schematic representation of GEF architecture: (**A**) Representative GEFs for the Ras, Rab, Arf, Ran, and Rho families are shown. Domains are defined by SMART (http://smart.embl-heidelberg.de) or Pfam (https://pfam.xfam.org/). The pink and green bars depict low complexity and coiled coils, respectively. (**B**) Molecular phylogenetic analysis of GEFs by the maximum likelihood method. Evolutionary analyses were conducted in MEGA7 [19]. GEF domains have been aligned to produce this phylogenetic tree.

GEFs show diverse structures although they have conserved activity mechanisms (Figure 2). However, GEFs must have some specificity for one or more cognate small GTPases to enable precise signaling. The first complex structure reported was RALGDS:H-Ras in 1988. This structure contains a Mg^2+^ ion and a phosphoaminophosphonic acid-guanylate ester (GNP) [17]. In the same year, the SOS1:H-Ras structure without nucleotides or Mg^2+^ ions was reported [18]. To date, over 300 GEF structures have been determined. Among them, 133 structures are GEF:small GTPase complex structures (Table 1). We next summarize the GEF mechanism based on structural work.

### 2.2. Common Mechanisms of GEF-Stimulated Exchange Reactions

**Figure 3:**
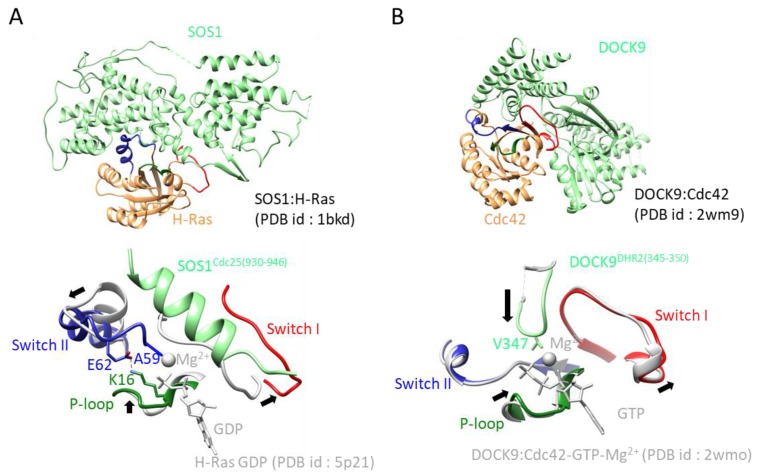
GEF:GTPase structures and their exchange mechanisms: (**A**) Upper: overall structure of the SOS1:H-Ras complex. SOS1 and H-Ras are colored green and orange, respectively. The P-loop, switch I, and switch II are colored green, red, and blue, respectively. Lower: Close up view of the active site of the SOS1:H-Ras complex: structural rearrangement is indicated by a black arrow. Key residues for exerting GEF activity are shown as stick models. The color scheme of the complex follows that of upper figure. The structure of the GDP-bound form of H-Ras, including GDP and Mg^2+^, is overlaid and colored in gray. (**B**) Upper: overall structure of the Dock9:Cdc42 complex. The structure of the GDP-bound form of Cdc42, including GTP and Mg^2+^, is overlaid. Lower: Close up view of the active site of the Dock9:Cdc42 complex. The color scheme and other descriptions follow those of Figure 1B.

The consensus function of the GEF is to stimulate nucleotide-exchange reactions by forming complexes with cognate small GTPases. GEF reactions involve multiple steps. First, a GEF forms a low-affinity complex with a GDP-bound small GTPase and then forms a high affinity complex with nucleotide-free small GTPase. By binding GTP to small GTPases, the stable GTPase complex is disrupted, and eventually, the GTP-bound form is produced [4,5]. Unlike small GTPases, GEFs exhibit a much diversity, although the following structural studies indicated that there are some common GEF mechanisms. (1) Many GEFs first bind to the switch I region, move the switch region away by regions of steric hindrance, and then bind to the switch II region by forming a stable GEF:GTPase complex. The conformational changes of both switches release GDP form GEF. (2) Some GEFs insert an acidic residue into the phosphate-binding site to expel the bound nucleotide by electrostatic repulsion while other GEFs insert a hydrophobic residue near the Mg^2+^ binding site to expel Mg^2+^, thus reducing the binding affinity of a small GTPase for GTP. Finally, some GEFs use a conserved Ala residue on the switch region instead of a hydrophobic residue of their own (Figure 3).

### 2.3. Structures and Mechanisms of Family Specific GEFs

Next, we will divide the GEFs into six groups and describe their structural features, GEF mechanism, family specificity, and recent structural work focusing on GEF:small GTPase structures.

#### 2.3.1. The Cdc 25 Family; GEFs for Ras, Ral, Rap, and Roc

**Figure 4:**
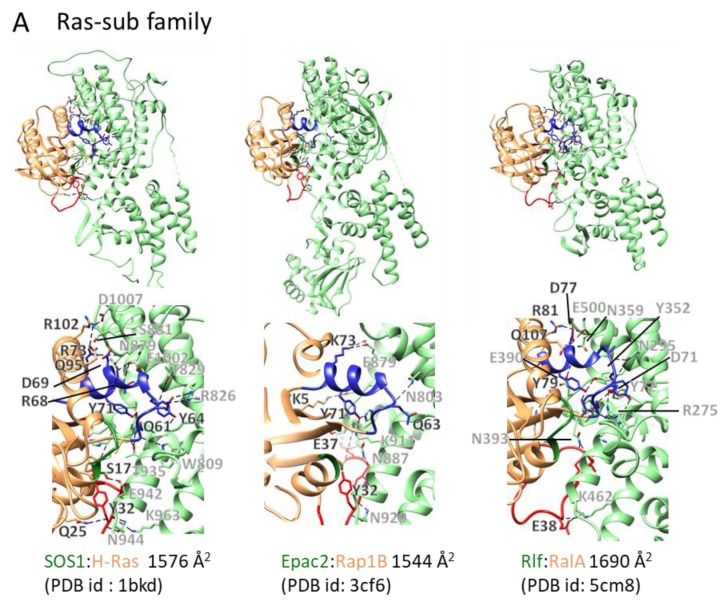

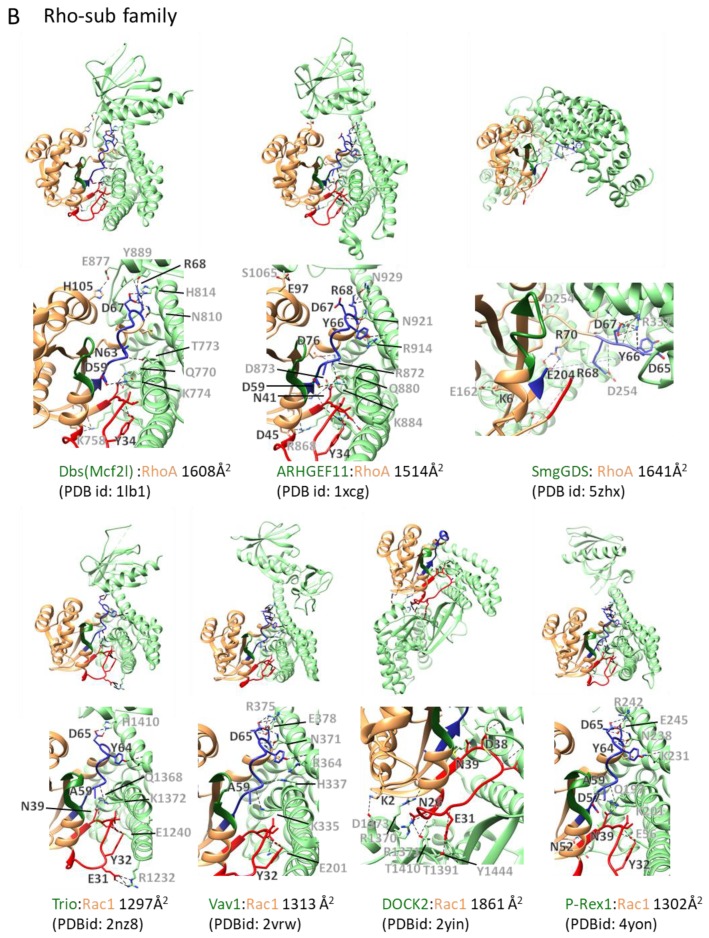

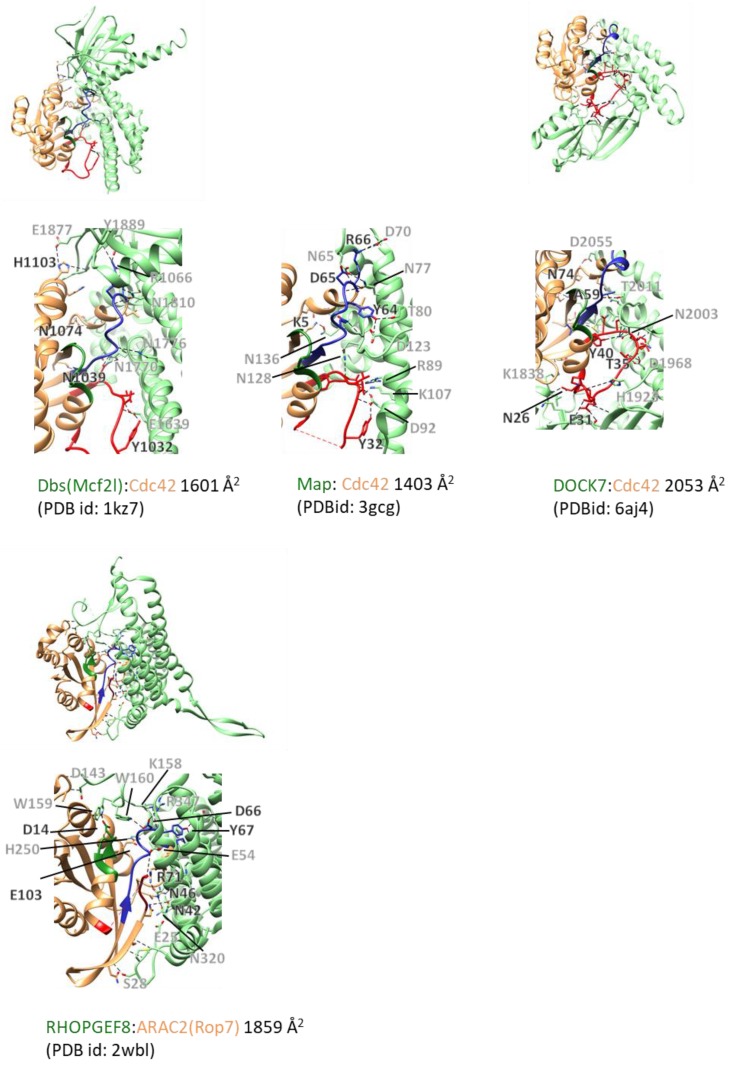

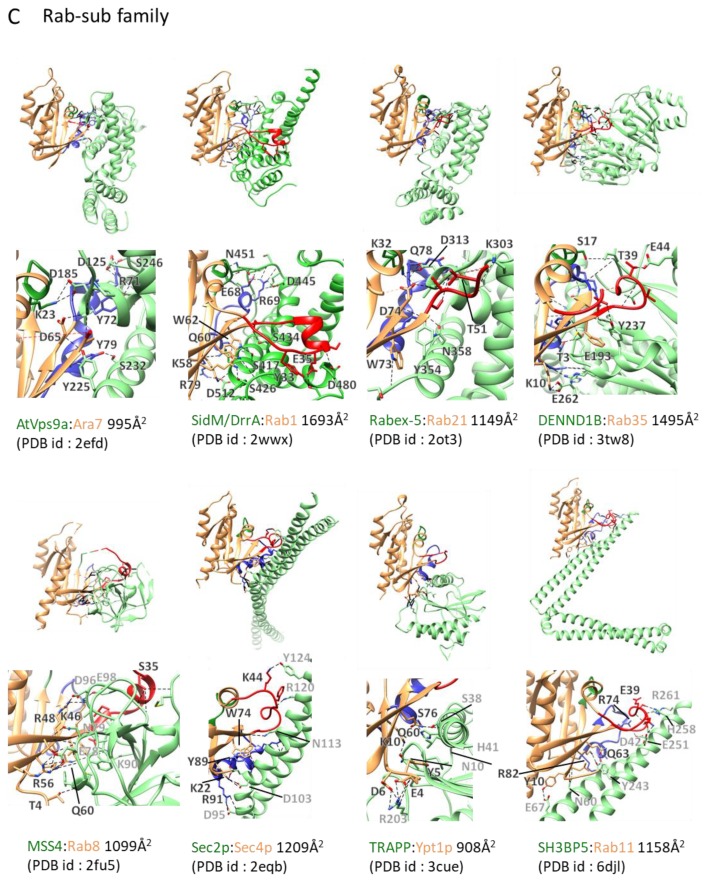

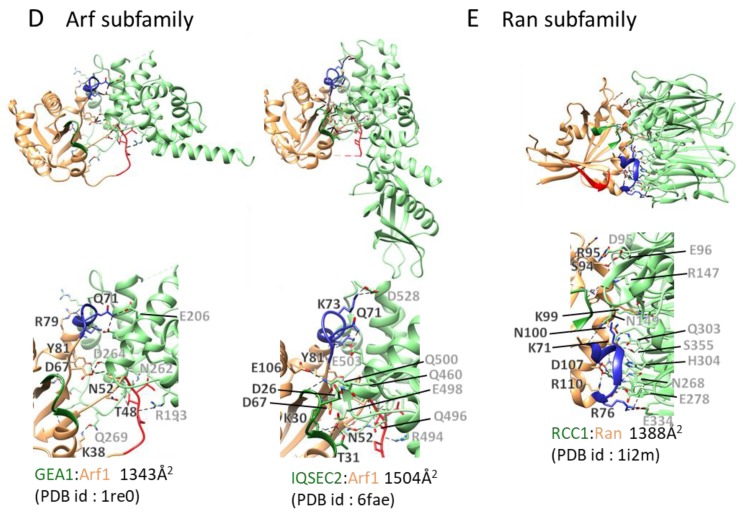
GEF:small GTPase complex structures and their interacting interface: GEF:small GTPase complex structures in the ligand unbound forms were drawn. The color scheme and other descriptions follow those of Figure 1B. Dashed lines depict hydrogen bonds or electrostatic interactions. The interaction areas and Protein Data Bank (PDB) ids are shown. (**A**) Ras subfamily. (**B**) Rho subfamily. (**C**) Rab subfamily. (**D**) Arf subfamily. (**E**) Ran subfamily.

Cdc25 homology domains (CDC25-HDs) are conserved from yeast to humans and function as GEFs for Ras, Ral, Rap, and Roc [20,21]. CDC25-HDs associate with the Ras exchange motif (REM). In 1998, a SOS1:H-Ras complex structure was reported (Figure 4A), which is the first reported structure of a nucleotide-free small GTPase in complex with a GEF [18]. REM and CDC25-HD of SOS1 contain 200 residues and 300 residues, respectively. In addition, the SOS1:H-Ras structure showed that both domains consist of only α-helices and that CDC25-HD only interacts with Ras. Ten years after this initial structure, a second complex structure was reported consisting of EPAC and Rap1B. The structures of both the Ras:SOS1 complex and EPAC:Rap1B complex have been well studied, revealing the common exchange mechanism of CDC25-HD. CDC25-HD causes structural changes on both switches, including the movement of the switch I region away from the GDP-binding pocket via steric hindrance and by rearranging the switch II region. In this rearrangement, the Ala residue on the switch II region inserts near the Mg^2+^ binding position, and the Glu residue on the switch II region makes a salt bridge with Lys residue on the P-loop (Figure 3A) [18].

CDC25-HD-containing proteins only act as GEFs for the Ras subfamily proteins Ras, Rap, and Ral. Based on their specificity, these GEFs are classified as either Ras-GEF, Rap-GEF, or Ral-GEF. For example, SOS1, EPAC, and Rlf are considered a Ras-GEF, a Rap-GEF, and a Ral-GEF, respectively. No structures of complexes had been determined for a while after the structure of the EPAC:Rap1B complex was determined (Figure 4A). However, recently, three more complex structures have been reported: Rlf:RalA [22], RasGRP4:H-Ras [23], and RasGRP2:Rap1B [23]. These three complexes showed that CDC25-HD similarly binds its cognate small GTPase and that RasGRP4 and RasGRP2 exhibit different specificities although they are homologous proteins. RacGRP4 is specific for Ras, whereas RacGRP2 is specific for Rap1B. This structural work also clearly shows the residues that confer this specificity. A comprehensive mutagenesis-based study based on structural information identified several candidate amino acids on small GTPases. For example, E54, T61, and L70 on Rap are highly conserved amongst Rap proteins and appear to be involved in EPAC interactions. Mutations at these sites yielded lower activation by EPAC, and EPAC could not increase GEF activity for the variant H-Ras^D54E^, ^Q61T^, ^Q70L^ [24]. These mutational studies thus indicate that additional residues must contribute to the selectivity of the interactions between GEFs and G-proteins and suggest that comprehensive mutational analysis based on complex structures is required to unveil specificity.

During the past two years, 25 structures of complexes including either SOS1 with H-Ras or K-Ras have been deposited into the PDB (Table 1). These two Ras proteins are well known as major oncogenes. The direct inhibition of Ras proteins is extremely challenging because they bind GTP with picomolar affinity and have no additional pockets for regulating their activity. In addition, the binding surfaces between Ras and its regulators (GEFs, GAPs, and others) are usually flat, thus research for finding an inhibitor to disrupt Ras-GEF interactions has focused on SOS1. For suppressing RAS-dependent signaling, several distinct series of small molecule activators that bind to a hydrophobic pocket in SOS1 in a RAS-SOS1-RAS ternary complex were discovered. Some of these binders activated the SOS1-mediated nucleotide exchange, resulting in reduced RAS signaling through negative feedback on SOS1 [25,26,27]. Most recently, the inhibitor of SOS1 was reported [28]. These inhibitors block reloading of K-Ras with GTP by preventing formation of the K-Ras–SOS1 complex. The inhibitor binds into a surface pocket on SOS1, which is located immediately adjacent to the K-Ras binding site, similar to binding site of the activators.

#### 2.3.2. DH, Dock, PRONE, and SmgGDS; GEFs for Rho Proteins

Dbl homology (DH) domain, Dock, plant-specific Rop nucleotide exchanger (PRONE), and SmgGDS proteins work as GEFs for Rho subfamily proteins and structures as these proteins are not related to each other (Figure 2). Recently, 43 cognate small GTPase complex structures have been deposited in PDB, allowing for further analysis of these interactions (Figure 4B).

The first identified mammalian RhoGEF was Dbl [29,30], of which the amino acid sequence is homologous to that of *Saccharomyces cerevisiae* GEF Cdc24. The region of homology between them includes the ~200 amino residues termed the DH domain and the ~100 C-terminal residues termed the Pleckstrin homology (PH) domain. The DH domain folds into a helical bundle structure called the “chaise lounge” [31] and has many functions, including moving the switch I region; remodeling of the switch II region, including insertion of the Ala residue near the Mg^2+^ binding site; and stabilization of the P-loop by interacting Lys residues of the P-loop near the Glu residues on the switch II region (Figure 3B). A remarkable feature of all nucleotide-free complex structures involving GEFs is that the conserved Leu residue exits near the Mg^2+^ binding site. This Leu residue is therefore responsible for GEF activity.

DOCK proteins harbor the DHR1 and DHR2 domains, which are unrelated to the DH domain and are specific for Rac and Cdc42. The first complex structure involving DOCK consisted of the DHR2 domain of DOCK9 with Cdc42, published in 2009. The DHR2 domain consists of ~360 amino acid residues arranged into three lobes of roughly equal size (lobes A, B, and C) and possesses GEF activity. The Cdc42-binding site and catalytic center are generated entirely from lobes B and C [32].

Since 2009, the crystal structures of Dock1^DHR2^:Rac1 [33], Dock8^DHR2^:Cdc42 [34], and Dock7^DHR2^:Cdc42 [35] have been determined. The DHR2 domain moves the switch I region away from the nucleotide binding site and stabilizes it by forming specific interactions not seen in most other GEFs. The insertion of the hydrophobic Val residue in the flexible loop of GEFs near the Mg^2+^ binding site is also typically found (Figure 2B).

A protein containing a PRONE domain is the major RhoGEF family member in plants [36]. Structures of the PRONE domain of RopGEF8 in complex with either Rop7 or Rop4 have been reported [37,38]. The 365-amino-acid PRONE domain of RopGEF8 from *Arabidopsis thaliana* is almost entirely α-helical except for a β-turn formed by residues 84–104. PRONE is divided in two domains (subdomain 1 and 2). Although the PRONE structure is unrelated to that of other GEFs, the GEF mechanism is similar to that of other GEFs like SOS1. The PRONE domain has ionic interactions with the Lys residue on the P-loop and the Glu residue on switch II. Steric interfere with the Ala residue on the switch 2 region through positioning next to the Mg^2+^ site is also observed.

SmgGDS is an atypical GEF consisting only of armadillo-repeat motifs (ARMs). This protein works as both a GEF and a chaperone [37,39,40,41,42,43,44] and as a GEF can activate only RhoA and RhoC [40]. The SmugGDA protein was identified in 1990, but its crystal structure has only recently been reported [45,46]. The details of this structure are described in Section 2.3.3. 

The mechanism underlying the specificity of RhoGEF for Rho subfamily proteins has remained unclear. As for Cdc25-HD, comprehensive studies including structural analysis, mutagenesis-based experiments, and in vitro or in cell assays are required. Such studies for deciphering specificity have been attempted and described for Dbl proteins [47].

#### 2.3.3. Rab GEFs

Rabs are the largest branch of small GTPase superfamily, with more than 60 members in humans [48,49,50]. Their major role is in the regulation of vesicular traffic. In addition to their conserved structures consisting of general motifs, Rab proteins harbor several short insertion amino acid sequences termed RabF and RabSF [51,52], which are used for interacting with Rab-specific regulators (GEFs and GAPs). 

To date, many Rab GEFs have been identified [50]. The Vps9 and DENN families are two particularly large Rab families and consist of 9 members and 18 members in humans, respectively. These two families are unrelated and have different structures. In addition to these two families, several other unrelated Rab GEFs have been reported. Besides the low homology between Rab GEFs, there are many Rabs for which the cognate GEF has not yet been identified yet, although 23 GEFs that interact with 54 Rabs have been identified in human [50].

The Rab8:MSS4 structure, published in 2006, was first reported as a Rab:GEF complex structure. Compared to other GEF families, MSS4 is considered a chaperon rather than a GEF [53]. Since then, a variety of Rab:GEF crystal structures have been determined, including Sec4:Sec2 [54,55,56], Rab21:Rabex-5 [57], Ypt1p:TRAPP [58], Rab35:DennD1 [59], Rab8:GRAB/Rabin8 [60], and Rab5:Rabex-5 [61] (Figure 4C, Table 1). In all cases, RabGEFs bind to switch I and switch II regions and induce structural rearrangements to cause nucleotide release. The rearrangement enabling this release mainly occurs in the switch I region. Pulling the switch I region leads to displacement of a highly conserved aromatic Phe or Tyr residue in Rab proteins, as this Tyr residue forms an edge-to-face interaction with the guanine base [50]. In many cases, the region including this Tyr residue is moved away by GEFs and the electron density of this region is not been observed. In contrast to the switch I region, the switch II region exhibits an ordered conformation in complexes with GEFs and structural changes to switch II are not as drastic as those of the switch I region. In complexes with GEFs, the switch II conformation is more similar to the GTP-bound form than the GDP-bound form. Changes in P-loop structure are usually less drastic compared to those of the switch I and II regions. In addition to structural rearrangements of the switch regions, another common mechanism has been observed for nucleotide release, in which is the insertion of projection residues towards the Mg^2+^ binding site (Sec2:Sec4, Vps9:Ara7) or electrostatic repulsion effects between an acidic residue of a GEF and the phosphate group (Vps9, TRAPP).

Three complex structures involving Rabs have been deposited in the past two years. Two of these are of the same complex structure (SH3BP5:Rab11). Structural studies of SH3BP5 shows that its characteristic coiled-coil architecture mediates nucleotide exchange through its unique Rab-GEF interaction [62,63].

#### 2.3.4. GEFs for Arf, Arf-like, and Sar GTPases

Sar and Arf show unique conformational changes during the transition from inactive to active forms. GDP-bound Sar and Arf proteins exist in the cytosol and change their localization to the membrane upon structural transitions from inactive to active forms [4]. These characteristic structural changes couple membrane recruitment of Arf-family members with their activation by GEFs. Nine GEF domain:Arf complex structures have been deposited in the PDB (Table 1). The Arf GEFs harbor a conserved Sec7 catalytic domain consisting of ~200 amino acids that is composed of only α-helices, forming a superhelix [64]. Biochemical and crystallographic studies have provided a detailed mechanism for Arf activation (Figure 4D) [65]. First, the Sec7 domain recognizes the inactive Arf-GDP conformation and promotes the β-strand shift of the inter-switch, eliminating the binding pocket for the N-terminal helix and fixing Arf to the membrane prior to GDP dissociation. Next, GDP dissociation occurs. The Sec7 domain has a critical catalytic glutamate, termed the “glutamate finger”, of which the negative charge removes the nucleotide-bond before stabilizing the empty nucleotide-binding site by a salt bridge with the conserved P-loop lysine. Sar GTPase is activated on the endoplasmic reticulum by Sec12, which is conserved from yeast to mammals [66]. The nucleotide exchange activity is carried out by the 38 kDa cytoplasmic domain of Sec12, for which the structure was reported in 2012. It adopts a seven-bladed β propeller fold [67]. The whole structure resembles that of RanGEF RCC1, but its reaction mechanism has not been identified. For the Arf subfamily, a comprehensive study of specificity against cognate GTPase has not yet been reported.

#### 2.3.5. RanGEF

Ran is activated by a regulator of chromosome condensation 1 (RCC1). The crystal structure of Ran:RCC1 was reported in 2001, and since then, no complex structure with Ran has been reported. RCC1 consists of a seven-bladed β-propeller [68], and the Ran:RCC1 complex structure revealed that RCC1 binds to Ran using the top of the RCC1 β-propeller (Figure 4E). This interaction covers the switch II region and the α2 and α3 helices. In contrast to the rearrangement of switch I observed in other smallGTPase:GEF complexes, RCC1 slightly changes the conformation of the GDP-binding site and does not position stabilizing negative charges near the P-loop Lys or destabilizing hydrophobic residues near Mg^2+^, as found in other complexes. The mechanism of nucleotide release by RCC1 thus is unclear.

RCC1 is a single-domain protein; therefore, it is thought that it lacks autoregulation mechanisms. Instead, RCC1 interactions with feed-forward signaling effectors have been identified and well characterized. RCC1 binds directly to the nucleosome core particle, thus increasing its exchange efficiency [69]. Comparison of the structures of RCC1 in a complex with nucleotide-free Ran [68] and with the nucleosome [70] shows that Ran and its effector bind nonoverlapping regions of RCC1; thus, they can be simultaneously accommodated by the GEF.

#### 2.3.6. GEF for Mitochondrial Rho

The GEF for Mitochondrial Rho has not yet been identified. One strong candidate is Drosophila vimar, which shares sequence similarity with SmgGDS (RAP1GDS) [71]. The GEF activity of vimar in vitro has not yet been reported.

### 2.4. Higher Level Regulatory Mechanisms of GEFs

**Figure 5:**
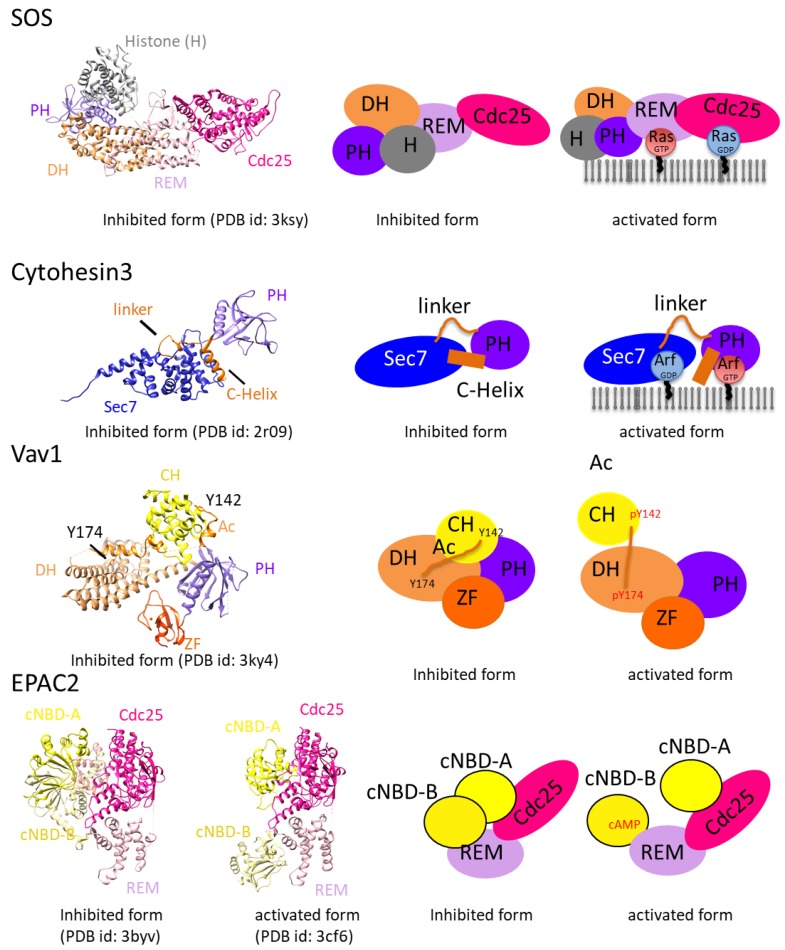
Higher level regulatory mechanisms of GEFs: Positive feedback loops of Rac/RasGEF SOS and Cytohesin3, regulation of Vav1 by the PH-DH module, and regulation of EPAC2 by the second messenger. The right panels show models for each regulatory mechanism.

Almost all GEFs are multidomain proteins that are regulated in a highly complex fashion (Figure 2), including through protein–protein interactions and the binding of second messengers. Additionally, autoregulation through intramolecular interactions is important for accepting and inducing precise signals. Several GEF structures are considered auto-inhibited structures because the GTPase binding site is hidden by a domain or motif that flanks the catalytic domain of the GEF and because large domain rearrangement must occur for full GEF activity. Additionally, activated GTPases can bind to GEFs and modify the GTP/GDP exchange reaction in a positive-feedback loop that is a key regulatory modality for amplifying an initial burst of activating signal (Figure 5).

#### 2.4.1. The Feedback Loop of Rac/RasGEF SOS

Structural studies of the abovementioned positive-feedback loop have been carried out on Rac/RasGEF SOS. The GTP-bound form of the GTPase, the product of the exchange reaction, binds to the GEF and modifies its basal exchange rate. SOS is comprised of a histone-like domain, a DH-PH tandem that activates Rac downstream of Ras, a REM-Cdc25 tandem that activates Ras, and a C-terminal domain that binds the Grb2 adaptor. SOS1 has an allosteric Ras-GTP binding site located on the REM domain [72], which is blocked in auto-inhibited Ras by its DH domain which is auto-inhibited by its tandem PH domain, revealing that SOS1 has multilayer auto-inhibitory mechanisms [73,74]. These structural studies thus help describe the positive-feedback mechanism described above as a freshly formed Ras-GTP pair can bind to the allosteric site on the REM domain, thus inducing displacement of the DH domain and consequently relieving the auto-inhibited state.

#### 2.4.2. The Feedback Loop of ArfGEF

Allosteric activation of ArfGEFs by a positive-feedback loop has been reported for ArfGEF cytohesins, which harbor a Sec7 domain and a PH domain [75]. Cytohesins are auto-inhibited by the linker between their Sec7 and PH domains and the α-helix that follows their PH domain, which both cover the Arf binding site [76]. This allosteric activation mechanism was revealed by the structure of the C-terminal region of ArfGEF cytohesin-3, including structures of a PH domain in a complex with the head group of phosphatidyl inositol 3,4,5-trisphosphate and a GTP-bound Arf6 [77]. This study reveals that auto-inhibitory and membrane-targeting elements are required for Arf6-GTP binding. The relieving mechanism for this auto-inhibition was also revealed using structural information. In the past 2 years, only one complex structure with an ArfGEF, the SEC7 domain of IQSEC2 with ARF1, has been deposited in the PDB. This structure has been used to attempt to identify an inhibitor [59].

#### 2.4.3. Regulation by PH-DH Module

Biochemical and structural studies have revealed that PH domains of DH-PH RhoGEFs have a variety of regulatory functions, including auto-inhibition, assistance in exchange reactions, targeting of RhoGEFs to phosphoinositide-containing membranes, and/or contributions to signaling specificity by binding to up- or downstream proteins in the signaling pathway [78]. These roles are achieved by the structural flexibility between DH and PH domains. In many structures, the PH domain appears to inhibit the DH domain by steric hindrance. For example, in SOS, the PH domain obstructs the Rac GTPase-binding site of the DH domain [73,74,79], thus revealing why the RacGEF activity of SOS is auto-inhibited [80]. Conversely, it has been reported that the PH domains of various GEFs (Dbs, PDZ, LARG, and Trio) assist in nucleotide exchange to some degree [81,82,83]. Despite this work, the supporting mechanism of the PH domain has remained unclear. It is possible that PH domains contribute to nucleotide exchange by optimizing the orientation of the GEF domain relative to the membrane, as proposed for Tiam [84]. However, most assays for GEF activities are performed in solutions where membrane effects are not evaluated. The DH domain is also inhibited by the SH3 domain, as seen for the SH3 domain of Cdc42-GEF ASEF, which binds extensively to the DH catalytic site [85,86]. Detailed structural studies were performed using RacGEF Vav [87]. The conformation of auto-inhibited Vav was determined using single particle cryo-electron microscopy of full- length Vav3 [88], X-ray crystallography [89], and NMR [89,90,91,92]. The triggering events of auto-inhibition are the sequential phosphorylation of Tyr142, Tyr160, and Tyr174 in the acidic region of Vav upon receptor stimulation. Kinetics studies have also shown that RhoGEFs of the Tim subfamily are auto-inhibited by a phosphorylation motif [93] and the interaction of their C-terminal SH3 domain with an N-terminal polyproline motif [94]. RhoGEFs harboring an RGS domain are auto-inhibited by their RGS domain [95,96] and by a negatively charged patch located upstream of the DH domain [20]. Auto-inhibitory regulation has been reported for DOCK and Prone RhoGEFs [97,98,99,100], but the structural details have not reported yet.

Among DH-containing RhoGEFs, positive feedback regulation by activated RhoA has only been found in the lymphoid blast crisis (LBC) family of RhoGEF [101,102]. The crystal structure of the PH domain of p190RhoGEF in a complex with activated RhoA shows that activated RhoA binds a hydrophobic interface consisting of β5, β6, and β7 [103]. A positive-feedback loop mediated by activated Cdc42 has also been reported for the Cdc42 GEF Dock11. Activated Cdc42 binds to the PH domain and stimulates GEF activity in cell extracts [104].

#### 2.4.4. Regulation of GEF by Second Messenger

EPAC activates Rap GTPases in response to an increase in the second messenger cAMP [105,106]. EPAC is the only GEF for which the conversion of the auto-inhibited conformation to the active conformation has been revealed in structural detail. A full-length EPAC structure showed that the two cAMP binding domains (cNBDs) blocked the Rap binding site and that the two domains reciprocally block their cAMP-binding sites. A structure of active cAMP-bound EPAC with Rap1 shows that the cNBD domain was truncated and indicates that the remaining cNBD domain has moved away from the Rap binding site.

## 3. Local Protein Unfolding and Refolding of Small GTPase Induced by Binding

### 3.1. An Overview

**Figure 6:**
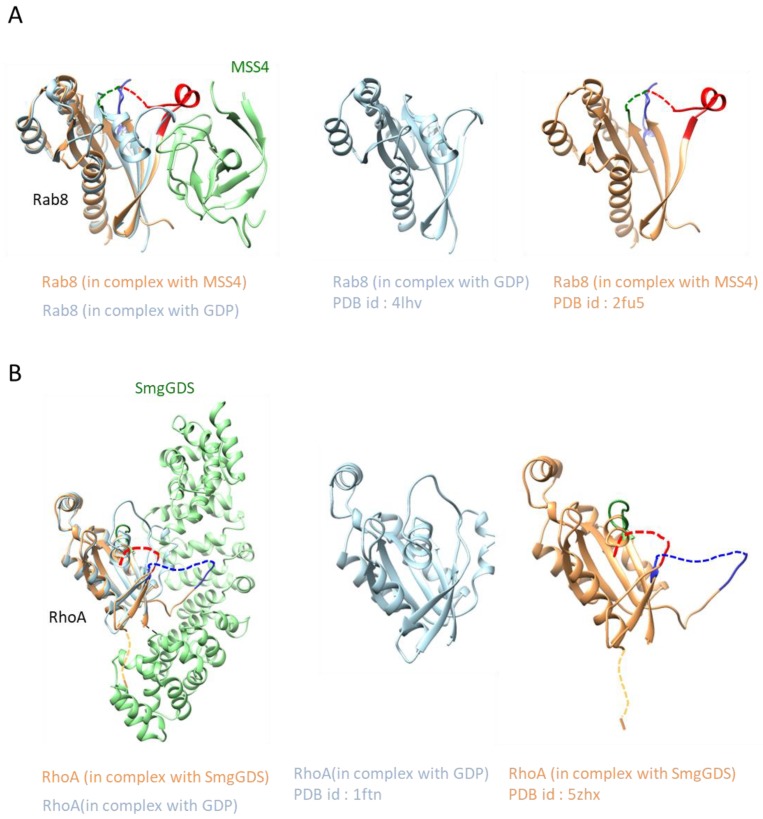
Local protein unfolding and refolding mechanism: (**A**) Structures of Rab8 in a complex with MSS4 and GDP-bound form and (**B**) Structures of RhoA in a complex with SmgGDS and GDP-bound form. The nucleotide recognition regions (P loop, switch I, and switch II) are almost ordered in the unbound form, but these regions are largely disordered in both proteins upon binding of the regulator. Disordered regions are shown as dashed lines. The color scheme and other descriptions follow those of Figure 1B.

In the above sections, we highlighted the well-established structural mechanisms of GEFs. Generally, GEF stabilization of nucleotide-free small GTPases is accompanied by structural rearrangements in the switch regions. Two intriguing structures, MSS4:Rab8 and SmgGDS:RhoA^farnesylated^, exhibit dramatic structural changes of the bound small GTPases, leading to local protein unfolding (Figure 6). In addition, both the MSS4 and SmgGDS proteins are known as chaperons in addition to their GEF function. In this section, we introduce the unfolding and refolding of GTPases by these regulators.

### 3.2. MSS4

MSS4 is reported to be a GEF for Rab proteins and shows chaperone activity for nucleotide-free Rab. MSS4 is involved in the exocytic pathway and helps stimulate neurotransmitters [107]. MSS4 GEF activity was shown in living cells [108]. Additionally, MSS4 is overexpressed in a wide variety of malignant tissues, including human pancreatic and colon cancers, suggesting a potential role in cancer progression via enhanced secretion of trophic factors required for tumor proliferation and maintenance [109]. It was pointed out that the GEF activity of MSS4 was low compared with other GEFs [110,111,112]. Some studies have also shown that DSS4, which is the yeast homologue of MSS4, and MSS4 could alleviate the harmful effects of Rab mutations [113,114,115]. These data thus indicate that DSS4/MSS4 may work as a chaperone, which was confirmed in a recent study showing that MSS4 mainly works as chaperon during GULUT4 exocytosis and has no function as a GEF [116].

The MSS4:Rab8 complex has a crescent shape in which the two molecules share a small interface (Figure 4C). The MSS4 molecule consists of three β-sheets composed of β strands βA to βL, the helices, 3_10_A and 3_10_B, and a single Zn finger motif. The entire structure of the MSS4:Rab8 complex is very similar to the previously determined MSS4 structure [117,118], but differences were observed in the flexible βE–βF and βH–βI loops. The βE–βF loop is largely disordered, while the βH–βI loop is involved in Rab8 binding [117,118]. The overall structure of Rab8 in a complex with MSS4 shows a typical small GTPase fold. However, significant differences compared to other known Rab structures were found. The crystal structure shows that nucleotide-free MSS4:Rab8 complex formation induces drastic structural changes of the Rab8 molecule, which are largely concentrated in regions involved in nucleotide and Mg^2+^ binding. MSS4 binds to the switch I and inter-switch regions of Rab8, resulting in a disordered nucleotide-binding pocket of Rab8 and displacement of residues involved in nucleotide interactions. The rigid MSS4 molecule stabilizes the nucleotide-free Rab8 through new interactions, and proper function of Rab8 requires a cycle of MSS4-induced GTPase unfolding and refolding and the release of MSS4.

### 3.3. SmgGDS

In a similar manner to MSS4, SmgGDS employs an unfolding and refolding strategy. SmgGDS functions as a GEF for Rho family proteins and as a chaperone for small GTPases with a CaaX motif [37,39,40,41,42,43,44]. SmgGDS interacts with various small GTPases possessing a C-terminal domain upstream of the CaaX motif in HVR [39,40,119,120,121] and is overexpressed in certain types of cancer cells, including breast cancer cells [122,123,124,125]. Among RhoGEFs, SmgGDS has unique architectures; is an entirely alpha-helical protein composed of armadillo-repeat motifs (ARMs) [126]; and has two splice variants, SmgGDS-558 and SmgGDS-607, which only vary in one insertion ARM (Figure 4A). These splice variants differ in GEF activity and binding affinity for RhoA depending on their prenylation state [42,43,46], as SmgGDS-558 prefers to bind prenylated RhoA while SmgGDS-607 favors nonprenylated RhoA [46]. The GEF activity mechanism underlying this activity has been long known; however, recently the crystal structures of SmgGDS:RhoA^farneylated^ were reported [45] and no guanine nucleotides or Mg^2+^ were observed. This suggests that the guanine and Mg^2+^ cofactors are released by SmgGDS-558 binding. PBR of RhoA exists on the negatively charged region formed by a concentration of acidic residues in the N-terminus. The switch II region of RhoA is bound by the switch II binding region in the middle of SmgGDS, which is positively charged in both the switch II region and its flanking region.

The most remarkable features of the complex structure are the large conformational changes in both the switch I and II regions. The switch I region is completely disordered and was not able to bind a nucleotide. SmgGDS-558 appears to have pulled out a part of switch II into the switch II binding region, resulting in a drastic conformational change accompanied by disruption of the α2-helix of RhoA. Such conformational changes in the switch I and II regions could disrupt important interactions among RhoA, Mg^2+^, and guanine and could facilitate guanine dissociation. Comparison of the SmgGDS-558/farnesylated RhoA structure to other complex structures of GEFs and small GTPases only indicates that SmgGDS-558 does not recognize switch I. Generally, other GEFs recognize switch I and II cooperatively. To date, over 35 structures of RhoA have been deposited into the Protein Data Bank, and 7 of them (PDB ID code 1LB1, 1 × 86, 1XCG, 3T06, 4XH9, 5JHG, and 5JHH) are guanine nucleotide and magnesium-free forms of RhoA. Notably, none of them are similar to the SmgGDS:RhoA^farneylated^ crystal structure.

In addition to its unique GEF mechanism, SmgGDS has a unique lipid recognition mechanism. SmgGDS-558 forms a cryptic pocket to accommodate the prenyl group of RhoA. A farnesyl group that modifies the CaaX motif is inserted into a cryptic pocket created between the ARM B and D regions.

Two structures of prenyl group-accommodating proteins for small GTPases, RhoGDI, and PDEδ have been determined. Both proteins shield the lipid group modifying small GTPases and sequester the small GTPases in the cytosol to protect them from aggregation and degradation or to enhance their diffusion in the cell [127,128,129]. SmgGDS-558 accommodates the prenyl group of RhoA to a hydrophobic pocket in a similar manner to RhoGDI and PDEδ, indicating that SmgGDS-558 can act as a chaperone. However, SmgGDS-558 shows significant differences in its structure and recognition mechanism. RhoGDI and PDEδ fold into similar immunoglobulin-like β sandwiches comprised of two antiparallel beta-sheets and catch the prenyl group in a hydrophobic pocket of the immunoglobulin-like fold [127,128,129]. The only evidence that SmgGDS-558 can recognize the prenyl group of RhoA suggests that SmgGDS-558 can only act as a chaperone of matured RhoA.

The CaaX motif of small GTPases is proposed to be posttranslationally modified in three steps. In the first step, the cysteine residue of the CaaX motif is prenylated by FTase or GGTase in the cytosol. Next, at the ER membrane, the three terminal residues of CaaX are removed by the Ras converting enzyme RCE1 following methylation of the prenylated-cysteine residue by isoprenylcysteine methyl transferase (ICMT) [130,131]. These modifications are essential for small GTPases to target them to their correct location and for proper function. Further studies are required to clarify the influence of the earlier two steps on an SmgGDS/RhoA interaction.

## 4. Conclusions

To date, 133 GEF:Ras superfamily small GTPase complex structures (Table 1) have been deposited in the PDB. These intensive structural studies have improved our understanding of the GEF mechanism. Some common principles of the GEF process have been established, but structural work with MSS4:Rab8 and SmgGDS:RhoA have provided new insights into the GEF mechanism. For example, the “unfolding and refolding” strategy can be considered the second category of the GEF mechanism. Additionally, there are many small GTPases of which the GEF mechanism has not been identified yet. These unidentified GEFs and their mechanisms still need to be unveiled. Moreover, our knowledge of GEF mechanisms should be expanded to fully understand different physiological states.

## Figures and Tables

**Table 1:** GEF:Small GTPase structures.

No	PDB ID	Year	GEF	SmallG	GEF	Reso	Ligand *^1^	Disorder *^2^ (p,sw1,sw2)	HVR *^3^
1	1LFD	1998	RALGDS	H-Ras	RasGEF	2.1	GNP	no, no, no	no
2	1BKD	1998	SOS1	H-Ras	RasGEF	2.8		no, no, no	no
3	1NVU	2000	SOS1	H-Ras	RasGEF	2.2		no, no, no	no
4	1NVV	2003	SOS1	H-Ras	RasGEF	2.2	GNP	no, no, no	no
5	1NVW	2003	SOS1	H-Ras	RasGEF	2.7	GNP	no, no, no	no
6	1NVX	2003	SOS1	H-Ras	RasGEF	3.2	GTP	no, no, no	no
7	1XD2	2004	SOS1	H-Ras	RasGEF	2.7	GDP	no, no, no	no
8	3CF6	2008	Epac2	Rap1B	RapGEF	2.2	SO_4_	no, no, no	no
9	4NYJ	2014	SOS1	H-Ras	RasGEF	2.9	GNP	no, no, no	no
10	4NYM	2014	SOS1	H-Ras	RasGEF	3.6		no, no, no	no
11	4MGI	2014	Epac	Rap1b	RapGEF	2.8	SO_4_	no, no, no	no
12	4MGK	2014	Epac	Rap1b	RapGEF	2.7	SO_4_	no, no, no	no
13	4MGY	2014	Epac	Rap1b	RapGEF	2.6	SO_4_	no, no, no	no
14	4MGZ	2014	Epac	Rap1b	RapGEF	3.0	SO_4_	no, no, no	no
15	4MH0	2014	Epac	Rap1b	RapGEF	2.4	SO_4_	no, no, no	no
16	4URU	2015	SOS1	H-Ras	RasGEF	2.8		no, no, no	no
17	4URV	2015	SOS1	H-Ras	RasGEF	2.6		no, no, no	no
18	4URW	2015	SOS1	H-Ras	RasGEF	2.8		no, no, no	no
19	4URX	2015	SOS1	H-Ras	RasGEF	2.5		no, no, no	no
20	4URY	2015	SOS1	H-Ras	RasGEF	2.5		no, no, no	no
21	4URZ	2015	SOS1	H-Ras	RasGEF	2.2		no, no, no	no
22	4US0	2015	SOS1	H-Ras	RasGEF	2.2		no, no, no	no
23	4US1	2015	SOS1	H-Ras	RasGEF	2.7	L71	no, no, no	no
24	4US2	2015	SOS1	H-Ras	RasGEF	2.5	L71	no, no, no	no
25	5CM8	2015	Rlf (Rgl2)	RalA	RalGEF	2.6		no, no, no	no
26	6AXG	2017	RasGRP4	H-Ras	RasGEF	3.3		no, yes, no	no
27	6AXF	2017	RasGRP	Rap1b	RapGEF	3.1		no, no, no	no
28	6D55	2018	SOS1	H-Ras	RasGEF	1.7	Na, FMT, GOL, Mg, GNP, FWA	no, no, no	no
29	6D5W	2018	SOS1	H-Ras	RasGEF	2.5	FVV, Mg, GNP	no, no, no	no
30	6D56	2018	SOS1	H-Ras	RasGEF	1.7	Na, FMT, GOL, Mg, GNP, FVN	no, no, no	no
31	6D59	2018	SOS1	H-Ras	RasGEF	1.7	Na, FMT, GOL, Mg, GNP, FVJ	no, no, no	no
32	6D5E	2018	SOS1	H-Ras	RasGEF	1.8	Na, FMT, GOL, Mg, GNP, FVG, CL	no, no, no	no
33	6D5G	2018	SOS1	H-Ras	RasGEF	1.9	FMT, GOL, Mg, GNP, FVD, Cl, BME	no, no, no	no
34	6D5H	2018	SOS1	H-Ras	RasGEF	1.8	FMT, GOL, MG, GNP, FV7, Cl	no, no, no	no
35	6D5J	2018	SOS1	H-Ras	RasGEF	1.8	Na, FMT, GOL, MG, GNP, FV4	no, no, no	no
36	6D5L	2018	SOS1	H-Ras	RasGEF	1.7	Na, FMT, GOL, MG, GNP, FW7	no, no, no	no
37	6D5V	2018	SOS1	H-Ras	RasGEF	2.0	Mg, GNP, FVY	no, no, no	no
38	6D5M	2018	SOS1	H-Ras	RasGEF	2.1	Mg, GNP, FW4	no, no, no	no
39	5WFO	2018	SOS1	H-Ras	RasGEF	2.0	Mg, GNP, 5UU	no, no, no	no
40	5WFP	2018	SOS1	H-Ras	RasGEF	2.1	Mg, GNP, 5UX	no, no, no	no
41	5WFQ	2018	SOS1	H-Ras	RasGEF	2.3	Mg, GNP, 5UV	no, no, no	no
42	5WFR	2018	SOS1	H-Ras	RasGEF	2.5	Mg, GNP, 5UW	no, no, no	no
43	6BVI	2018	SOS1	H-Ras	RasGEF	1.8	NA, FMT, EC4, GOL, Mg, GNP	no, no, no	no
44	6BVJ	2018	SOS1	H-Ras	RasGEF	1.7	NA, FMT, EAS, GOL, Mg, GNP	no, no, no	no
45	6BVK	2018	SOS1	H-Ras	RasGEF	1.8	NA, FMT, EAV, GOL, Mg, GNP	no, no, no	no
46	6BVL	2018	SOS1	H-Ras	RasGEF	1.7	NA, FBY, EAV, GOL, Mg, GNP	no, no, no	no
47	6BVM	2018	SOS1	H-Ras	RasGEF	1.8	NA, FBY, EBV, GOL, Mg, GNP	no, no, no	no
48	6EPL	2019	SOS1	K-Ras	RasGEF	2.6	GOL	no, no, no	no
49	6EPM	2019	SOS1	K-Ras	RasGEF	2.5	BQ5, GOL	no, no, no	no
50	6EPN	2019	SOS1	K-Ras	RasGEF	2.5	BQ2, DMS, GOL	no, no, no	no
51	6EPO	2019	SOS1	K-Ras	RasGEF	2.4	GOL, BPW	no, no, no	no
52	6EPP	2019	SOS1	K-Ras	RasGEF	2.4	GOL, BOQ	no, no, no	no
53	1LB1	2002	Dbs (Mcf21)	RhoA	RhoGEF	2.8		no, no, no	no
54	1 × 86	2004	ARHGEF12	RhoA	RhoGEF	3.2	PO_4_	no, no, no	no
55	1XCG	2004	ARHGEF11 (PDZ-rhoGEF)	RhoA	RhoGEF	2.5		no, no, no	no
56	2RGN	2007	ARHGEF25 (p63RhoGEF)	RhoA	RhoGEF	3.5		no, no, no	no
57	3KZ1	2010	ARHGEF11 (PDZ-rhoGEF)	RhoA	RhoGEF	2.7	GSP	no, no, no	no
58	3LW8	2010	IpgB2	RhoA	RhoGEF	1.9	GDP	no, no, no	no
59	3LWN	2010	IpgB2	RhoA	RhoGEF	2.3	GDP	no, no, no	no
60	3LXR	2010	IpgB2	RhoA	RhoGEF	1.7	GDP	no, no, no	no
61	3T06	2011	ARHGEF11 (PDZ-rhoGEF)	RhoA	RhoGEF	2.8		no, no, no	no
62	4D0N	2014	AKAP13	RhoA	RhoGEF	2.1	GDP	no, no, no	no
63	4XH9	2015	NET1 (ARHGEF8)	RhoA	RhoGEF	2.0		no, yes, no	no
64	6BC0	2017	ARHGEF28 (p190RhoGEF)	RhoA	RhoGEF	2.2	GSP	no, no, no	no
65	5JHG	2017	ARHGEF11	RhoA	RhoGEF	2.5	GOL	no, no, no	no
66	6BCA	2017	ARHGEF18 (LbcRhoGEF)	RhoA	RhoGEF	2.0	GSP	no, no, no	no
67	6BCB	2017	ARHGEF18 (p114RhoGEF)	RhoA	RhoGEF	1.4	GSP	no, no, no	no
68	5JHH	2017	ARHGEF11	RhoA	RhoGEF	2.3	RAO	no, no, no	no
69	5ZHX	2018	SmgGDS	RhoA	RhoGEF	3.5		yes, yes, yes	yes
70	1FOE	2000	Tiam	Rac1	RhoGEF	2.8	SO_4_	no, no, no	no
71	2NZ8	2007	Trio	Rac1	RhoGEF	2.0		no, no, no	no
72	2VRW	2008	Vav1	Rac1	RhoGEF	1.9		no, no, no	no
73	2YIN	2011	DOCK2	Rac1	RhoGEF	2.7		no, no, no	no
74	3B13	2012	DOCK2	Rac1	RhoGEF	3.0		no, no, no	no
75	3BJI	2007	Vav1	Rac1	RhoGEF	2.6		no, no, no	no
76	4YON	2015	P-Rex1	Rac1	RhoGEF	2.0		no, no, no	no
77	5FI0	2016	P-Rex1	Rac1	RhoGEF	3.3		no, no, no	yes
78	5O33	2017	Kalirin	Rac1	RhoGEF	1.6	GDP	no, no, no	no
79	6BC1	2018	ARHGEF28 (p190RhoGEF)	Rac1	RhoGEF	2.9	GSP	no, no, no	no
80	1KZ7	2002	Dbs (Mcf2l)	Cdc42	RhoGEF	2.4		no, no, no	no
81	1GZS	2002	SOPE	Cdc42	RhoGEF	2.3	PO_4_	no, no, no	no
82	1KI1	2002	ITSN1	Cdc42	RhoGEF	2.3	PO_4_	no, no, no	no
83	1KZG	2002	Mcf2l (Dbs)	Cdc42	RhoGEF	2.6		no, no, no	no
84	2DFK	2006	ARHGEF9 (Collybistin II)	Cdc42	RhoGEF	2.2	PO_4_	no, no, no	yes
85	2WM9	2009	DOCK9	Cdc42	RhoGEF	2.2	GOL	no, no, no	no
86	2WMN	2009	DOCK9	Cdc42	RhoGEF	2.4	GDP	no, no, no	no
87	2WMO	2009	DOCK9	Cdc42	RhoGEF	2.2	GDP	no, no, no	no
88	3GCG	2009	map (L0028)	Cdc42	RhoGEF	2.3		no, yes, no	no
89	3QBV	2012	ITSN1	Cdc42	RhoGEF	2.7	GDP	no, yes, no	no
90	3VHL	2012	DOCK8	Cdc42	RhoGEF	2.1	PO_4_	no, no, no	no
91	6AJ4	2019	DOCK7	Cdc42	RhoGEF	3.2		no, no, no	yes
92	6AJL	2019	DOCK7	Cdc42	RhoGEF	3.2		no, no, no	yes
93	3CX6	2008	ARHGEF11 (PDZRhoGEF)	Galpha-13	RhoGEF	2.5	GDP	no, no, no	no
94	3CX7	2008	ARHGEF11 (PDZRhoGEF)	Galpha-13	RhoGEF	2.3	GSP	no, no, no	no
95	3CX8	2008	ARHGEF11 (PDZRhoGEF)	Galpha-13	RhoGEF	2.5	GDP	no, no, no	no
96	1SHZ	2005	ARHGEF1 (p115RhoGEF)	Gnai1,13	RhoGEF	2.9	GDP+ALF	no, no, no	no
97	2NTY	2007	ROPGEF8	ROP4	RopGEF	3.1	GDP	no, yes, no	no
98	2WBL	2009	ROPGEF8	ROP7	RopGEF	2.9		no, yes, no	no
99	2WWX	2009	SidM/DrrA	Rab1	RabGEF	1.5		no, no, no	no
100	3L0I	2010	SidM/DrrA	Rab1	RabGEF	2.9	SO_4_	no, no, no	no
101	3JZA	2010	DrrA/SidM	Rab1b	RabGEF	1.8	PO_4_	no, no, no	no
102	5O74	2017	DrrA/SidM	Rab1b	RabGEF	2.5	GDP	no, yes, yes	no
103	2OT3	2007	Rabex-5 (RabGEF1)	Rab21	RabGEF	2.1		no, yes, no	no
104	3TW8	2011	DENND1B	Rab35	RabGEF	2.1		no, yes, no	no
105	4Q9U	2014	Rabex-5 (RabGEF1)	Rab5A	RabGEF	4.6		yes, yes, no	no
106	2FU5	2006	MSS4	Rab8A	RabGEF	2.0		no, yes, yes	no
107	4LHX	2013	RAB3IP (Rabin8)	Rab8A	RabGEF	3.1	SO_4_	no, no, no	no
108	4LHY	2013	RAB3IP (Rabin8)	Rab8A	RabGEF	3.1	GDP	no, no, no	no
109	4LHZ	2013	RAB3IP (Rabin8)	Rab8A	RabGEF	3.2	GTP	no, no, no	no
110	4LI0	2013	RAB3IP (Rabin8)	Rab8A	RabGEF	3.3	GDP	no, no, no	no
111	2EFD	2010	AtVps9a	Ara7 (RabF2B)	RabGEF	3.0		no, yes, no	no
112	2EFE	2010	AtVps9a	Ara7 (RabF2B)	RabGEF	2.1	GDP	no, no, no	no
113	2EFH	2010	AtVps9a	Ara7 (RabF2B)	RabGEF	2.1	GDP	no, yes, no	no
114	2EFC	2010	AtVps9a	Ara7 (RabF2B)	RabGEF	2.1	GDP	no, no, no	no
115	4G01	2013	Vps9a	Ara7 (RabF2B)	RabGEF	2.2	GDP	no, no, no	no
116	2OCY	2007	Sec2p	Sec4p	RabGEF	3.3	GDP	no, no, no	no
117	2EQB	2007	Sec2p	Sec4p	RabGEF	2.7		no, no, no	no
118	4ZDW	2015	Sec2p	Sec4p	RabGEF	2.9		no, no, no	no
119	3CUE	2008	TRAPPI assembly	Ypt1p	RabGEF	3.7		no, yes, no	no
120	5LDD	2017	Mon1-Ccz1	Ypt7	RabGEF	2.5	SO_4_	no, no, no	no
121	6IXV	2019	SH3BP5	Rab11	RabGEF	3.8	PO_4_	no, no, no	no
122	6EKK	2019	DENND 1A	Rab35	RabGEF	1.8	GDP, SO_4_, EDO	no, no, no	no
123	6DJL	2019	SH3BP5	Rab11	RabGEF	3.1		no, no, no	no
124	1RE0	2003	GEA1	ARF1	ArfGEF	2.4	GDP	no, no, no	no
125	1R8Q	2003	CYTH2 (Arno)	ARF1	ArfGEF	1.9	G3D	no, no, no	no
126	1R8S	2003	CYTH2 (Arno)	ARF1	ArfGEF	1.5	GDP	no, no, no	no
127	1S9D	2003	CYTH2 (Arno)	ARF1	ArfGEF	1.8	GDP	no, yes, no	no
128	4C0A	2013	IQSEC1	ARF1	ArfGEF	3.3	G3D	no, no, no	no
129	6FAE	2018	IQSEC2	ARF1	ArfGEF	2.3		no, yes, no	no
130	4KAX	2013	CYTH3	ARF6	ArfGEF	1.9	GTP	no, no, no	no
131	5EE5	2016	ARFGEF1	ARL1	ArfGEF	2.3	GTP	no, no, no	no
132	5J5C	2016	ARFGEF1	ARL1	ArfGEF	3.4	GTP	no, no, no	no
133	1I2M	2001	RCC1	RAN	RanGEF	1.8	SO_4_	no, yes, no	no

*1 The names of the ligands are described below. GNP: phosphoaminophosphonic acid-guanylate ester; L71: (3S)-3-[3-(aminomethyl)phenyl]-1-ethylpyrrolidine-2,5-dione; FMT: formic acid; GOL: glycerol; FWA: 6-chloro-2-(2,6-diazaspiro [3.3]heptan-2-yl)-1-[(4-fluoro-3,5-dimethylphenyl)methyl]-4-(4-methylpiperazin-1-yl)-1H-benzimidazole; FVV: 10-[(4-fluorophenyl)methyl]-2,3,4,10-tetrahydropyrimido [1,2-a]benzimidazole; FVN: 6-chloro-2-(2,6-diazaspiro[3.3]heptan-2-yl)-4-(3,5-dimethyl-1H-pyrazol-4-yl)-1-[(4-fluoro-3,5-dimethylphenyl)methyl]-1H-benzimidazole; FVJ: 6-chloro-4-(3,5-dimethyl-1H-pyrazol-4-yl)-1-[(4-fluoro-3,5-dimethylphenyl)methyl]-2-(piperazin-1-yl)-1H-benzimidazole; FVG: 1-[(2S)-1-{6-chloro-1-[(4-fluoro-3,5-dimethylphenyl)methyl]-2-(piperazin-1-yl)-1H-benzimidazol-4-yl}pyrrolidin-2-yl]methanamine; FVD: 6-chloro-1-[(4-fluoro-3,5-dimethylphenyl)methyl]-2-(piperazin-1-yl)-4-(1,2,3,6-tetrahydropyridin-4-yl)-1H-benzimidazole; BME: beta-mercaptoethanol; FV7: 6-chloro-4-(2-chlorophenyl)-1-[(4-fluoro-3,5-dimethylphenyl)methyl]-2-(piperazin-1-yl)-1H-benzimidazole; FV4: 6-chloro-1-[(4-fluoro-3,5-dimethylphenyl)methyl]-2-(piperazin-1-yl)-1H-benzimidazole; FW7: 6-chloro-1-[(3-chloro-4-fluorophenyl)methyl]-2-(piperazin-1-yl)-1H-benzimidazole; FVY: 1-[(3-chloro-4-fluorophenyl)methyl]-5,6-dimethyl-1H-benzimidazol-2-amine; FW4: 1-[(3-chloro-4-fluorophenyl)methyl]-5,6-dimethyl-2-(piperazin-1-yl)-1H-benzimidazole; 5UU: 6-chloranyl-~{N}-(4-fluorophenyl)-1,2,3,4-tetrahydroacridin-9-amine; 5UX: 6-chloranyl-~{N}-(3-chloranyl-4-fluoranyl-phenyl)-1,2,3,4-tetrahydroacridin-9-amine; 5UV: 7-chloranyl-~{N}-(3-chloranyl-4-fluoranyl-phenyl)-1,2,3,4-tetrahydroacridin-9-amine; 5UW: ~{N}-(3,3-diphenylpropyl)piperidin-4-amine; EC4: 6-chloro-N-{1-[(5-chloro-1H-indol-3-yl)methyl]piperidin-4-yl}-L-tryptophanamide; EAS: 5-chloro-N-{1-[(5-chloro-1H-indol-3-yl)methyl]piperidin-4-yl}-L-tryptophanamide; EAV: N-{1-[(5-chloro-1H-indol-3-yl)methyl]piperidin-4-yl}-6-methyl-L-tryptophanamide; EBY: N-{1-[(5-chloro-1H-indol-3-yl)methyl]piperidin-4-yl}-5-methyl-L-tryptophanamide; EBV: (2S)-2-amino-1-[(3aR,6aS)-5-[(5-chloro-1H-indol-3-yl)methyl]hexahydropyrrolo[3,4-c]pyrrol-2(1H)-yl]-3-(1H-indol-3-yl)propan-1-one; BQ5: (1-phenyl-5,6-dihydro-4~{H}-cyclopenta[c]pyrazol-3-yl)methanamine; BQ2: 1-(3,4-dihydro-1~{H}-isoquinolin-2-yl)-2-oxidanyl-ethanone, DMS: dimethyl sulfoxide; BPW: 3-(4-chlorophenyl)propan-1-amine; BOQ: ethyl 2-(aminomethyl)-5-~{tert}-butyl-furan-3-carboxylate; GSP: 5′-guanosine-diphosphate-monothiophosphate; RA0: 3-{3-[ethyl(quinolin-2-yl)amino]phenyl}propanoic acid; ALF: tetrafluoroaluminate ion; EDO: 1,2-ethanediol; G3D: guanosine-3′-monophosphate-5′-diphosphate. *2 Disordered regions among P-loop (p), switch I (sw1), and switch II (sw2) are indicated. “yes” means that the region has a disordered structure. *3 The structure including the hyper variable region (HVR) is indicated as “yes”.

## References

[1] Wittinghofer A., Vetter I.R. (2011). Structure-function relationships of the G domain, a canonical switch motif. Ann. Rev. Biochem..

[2] Feinberg A.P., Vogelstein B., Droller M.J., Baylin S.B., Nelkin B.D. (1983). Mutation affecting the 12th amino acid of the c-Ha-ras oncogene product occurs infrequently in human cancer. Science.

[3] Vetter I.R., Wittinghofer A. (2001). The guanine nucleotide-binding switch in three dimensions. Science.

[4] Cherfils J., Zeghouf M. (2013). Regulation of small GTPases by GEFs, GAPs, and GDIs. Physiol. Rev..

[5] Bos J.L., Rehmann H., Wittinghofer A. (2007). GEFs and GAPs: Critical elements in the control of small G proteins. Cell.

[6] Reiss Y., Goldstein J.L., Seabra M.C., Casey P.J., Brown M.S. (1990). Inhibition of purified p21ras farnesyl: Protein transferase by Cys-AAX tetrapeptides. Cell.

[7] Seabra M.C., Reiss Y., Casey P.J., Brown M.S., Goldstein J.L. (1991). Protein farnesyltransferase and geranylgeranyltransferase share a common alpha subunit. Cell.

[8] Seabra M.C., Goldstein J.L., Sudhof T.C., Brown M.S. (1992). Rab geranylgeranyl transferase. A multisubunit enzyme that prenylates GTP-binding proteins terminating in Cys-X-Cys or Cys-Cys. J. Biol. Chem..

[9] Casey P.J., Seabra M.C. (1996). Protein prenyltransferases. J. Biol. Chem..

[10] Mitchell A.L., Attwood T.K., Babbitt P.C., Blum M., Bork P., Bridge A., Brown S.D., Chang H.Y., El-Gebali S., Fraser M.I. (2019). InterPro in 2019: Improving coverage, classification and access to protein sequence annotations. Nucleic Acids Res..

[11] Valencia A., Chardin P., Wittinghofer A., Sander C. (1991). The ras protein family: Evolutionary tree and role of conserved amino acids. Biochemistry.

[12] Walker J.E., Saraste M., Runswick M.J., Gay N.J. (1982). Distantly related sequences in the alpha- and beta-subunits of ATP synthase, myosin, kinases and other ATP-requiring enzymes and a common nucleotide binding fold. Embo J..

[13] Prive G.G., Milburn M.V., Tong L., de Vos A.M., Yamaizumi Z., Nishimura S., Kim S.H. (1992). X-ray crystal structures of transforming p21 ras mutants suggest a transition-state stabilization mechanism for GTP hydrolysis. Proc. Natl. Acad. Sci. USA.

[14] Rensland H., John J., Linke R., Simon I., Schlichting I., Wittinghofer A., Goody R.S. (1995). Substrate and product structural requirements for binding of nucleotides to H-ras p21: The mechanism of discrimination between guanosine and adenosine nucleotides. Biochemistry.

[15] Milburn M.V., Tong L., deVos A.M., Brunger A., Yamaizumi Z., Nishimura S., Kim S.H. (1990). Molecular switch for signal transduction: Structural differences between active and inactive forms of protooncogenic ras proteins. Science.

[16] Pai E.F., Kabsch W., Krengel U., Holmes K.C., John J., Wittinghofer A. (1989). Structure of the guanine-nucleotide-binding domain of the Ha-ras oncogene product p21 in the triphosphate conformation. Nature.

[17] Huang L., Hofer F., Martin G.S., Kim S.H. (1998). Structural basis for the interaction of Ras with RalGDS. Nat. Struct. Biol..

[18] Boriack-Sjodin P.A., Margarit S.M., Bar-Sagi D., Kuriyan J. (1998). The structural basis of the activation of Ras by Sos. Nature.

[19] Kumar S., Stecher G., Tamura K. (2016). MEGA7: Molecular Evolutionary Genetics Analysis version 7.0 for bigger datasets. Mol. Biol. Evol..

[20] Chardin P., Camonis J.H., Gale N.W., van Aelst L., Schlessinger J., Wigler M.H., Bar-Sagi D. (1993). Human Sos1: A guanine nucleotide exchange factor for Ras that binds to GRB2. Science.

[21] Crechet J.B., Poullet P., Mistou M.Y., Parmeggiani A., Camonis J., Boy-Marcotte E., Damak F., Jacquet M. (1990). Enhancement of the GDP-GTP exchange of RAS proteins by the carboxyl-terminal domain of SCD25. Science.

[22] Popovic M., Schouten A., Rensen-de Leeuw M., Rehmann H. (2016). The structure of the Guanine Nucleotide Exchange Factor Rlf in complex with the small G-protein Ral identifies conformational intermediates of the exchange reaction and the basis for the selectivity. J. Struct. Biol..

[23] Vercoulen Y., Kondo Y., Iwig J.S., Janssen A.B., White K.A., Amini M., Barber D.L., Kuriyan J., Roose J.P. (2017). A Histidine pH sensor regulates activation of the Ras-specific guanine nucleotide exchange factor RasGRP1. Elife.

[24] Popovic M., Rensen-de Leeuw M., Rehmann H. (2013). Selectivity of CDC25 homology domain-containing guanine nucleotide exchange factors. J. Mol. Biol..

[25] Abbott J.R., Hodges T.R., Daniels R.N., Patel P.A., Kennedy J.P., Howes J.E., Akan D.T., Burns M.C., Sai J., Sobolik T. (2018). Discovery of Aminopiperidine Indoles That Activate the Guanine Nucleotide Exchange Factor SOS1 and Modulate RAS Signaling. J. Med. Chem..

[26] Hodges T.R., Abbott J.R., Little A.J., Sarkar D., Salovich J.M., Howes J.E., Akan D.T., Sai J., Arnold A.L., Browning C. (2018). Discovery and Structure-Based Optimization of Benzimidazole-Derived Activators of SOS1-Mediated Nucleotide Exchange on RAS. J. Med. Chem..

[27] Burns M.C., Howes J.E., Sun Q., Little A.J., Camper D.V., Abbott J.R., Phan J., Lee T., Waterson A.G., Rossanese O.W. (2018). High-throughput screening identifies small molecules that bind to the RAS:SOS:RAS complex and perturb RAS signaling. Anal. Biochem..

[28] Hillig R.C., Sautier B., Schroeder J., Moosmayer D., Hilpmann A., Stegmann C.M., Werbeck N.D., Briem H., Boemer U., Weiske J. (2019). Discovery of potent SOS1 inhibitors that block RAS activation via disruption of the RAS-SOS1 interaction. Proc. Natl. Acad. Sci. USA.

[29] Eva A., Vecchio G., Rao C.D., Tronick S.R., Aaronson S.A. (1988). The predicted DBL oncogene product defines a distinct class of transforming proteins. Proc. Natl. Acad. Sci. USA.

[30] Hart M.J., Eva A., Evans T., Aaronson S.A., Cerione R.A. (1991). Catalysis of guanine nucleotide exchange on the CDC42Hs protein by the dbl oncogene product. Nature.

[31] Worthylake D.K., Rossman K.L., Sondek J. (2000). Crystal structure of Rac1 in complex with the guanine nucleotide exchange region of Tiam1. Nature.

[32] Yang J., Zhang Z., Roe S.M., Marshall C.J., Barford D. (2009). Activation of Rho GTPases by DOCK exchange factors is mediated by a nucleotide sensor. Science.

[33] Kulkarni K., Yang J., Zhang Z., Barford D. (2011). Multiple factors confer specific Cdc42 and Rac protein activation by dedicator of cytokinesis (DOCK) nucleotide exchange factors. J. Biol. Chem..

[34] Harada Y., Tanaka Y., Terasawa M., Pieczyk M., Habiro K., Katakai T., Hanawa-Suetsugu K., Kukimoto-Niino M., Nishizaki T., Shirouzu M. (2012). DOCK8 is a Cdc42 activator critical for interstitial dendritic cell migration during immune responses. Blood.

[35] Kukimoto-Niino M., Tsuda K., Ihara K., Mishima-Tsumagari C., Honda K., Ohsawa N., Shirouzu M. (2019). Structural Basis for the Dual Substrate Specificity of DOCK7 Guanine Nucleotide Exchange Factor. Structure.

[36] Mucha E., Fricke I., Schaefer A., Wittinghofer A., Berken A. (2011). Rho proteins of plants--functional cycle and regulation of cytoskeletal dynamics. Eur. J. Cell Biol..

[37] Thomas C., Fricke I., Scrima A., Berken A., Wittinghofer A. (2007). Structural evidence for a common intermediate in small G protein-GEF reactions. Mol. Cell.

[38] Thomas C., Fricke I., Weyand M., Berken A. (2009). 3D structure of a binary ROP-PRONE complex: The final intermediate for a complete set of molecular snapshots of the RopGEF reaction. Biol. Chem..

[39] Yamamoto T., Kaibuchi K., Mizuno T., Hiroyoshi M., Shirataki H., Takai Y. (1990). Purification and characterization from bovine brain cytosol of proteins that regulate the GDP/GTP exchange reaction of smg p21s, ras p21-like GTP-binding proteins. J. Biol. Chem..

[40] Mizuno T., Kaibuchi K., Yamamoto T., Kawamura M., Sakoda T., Fujioka H., Matsuura Y., Takai Y. (1991). A stimulatory GDP/GTP exchange protein for smg p21 is active on the post-translationally processed form of c-Ki-ras p21 and rhoA p21. Proc. Natl. Acad. Sci. USA.

[41] Hamel B., Monaghan-Benson E., Rojas R.J., Temple B.R., Marston D.J., Burridge K., Sondek J. (2011). SmgGDS is a guanine nucleotide exchange factor that specifically activates RhoA and RhoC. J. Biol. Chem..

[42] Berg T.J., Gastonguay A.J., Lorimer E.L., Kuhnmuench J.R., Li R., Fields A.P., Williams C.L. (2010). Splice variants of SmgGDS control small GTPase prenylation and membrane localization. J. Biol. Chem..

[43] Schuld N.J., Vervacke J.S., Lorimer E.L., Simon N.C., Hauser A.D., Barbieri J.T., Distefano M.D., Williams C.L. (2014). The chaperone protein SmgGDS interacts with small GTPases entering the prenylation pathway by recognizing the last amino acid in the CAAX motif. J. Biol. Chem..

[44] Williams C.L. (2013). A new signaling paradigm to control the prenylation and trafficking of small GTPases. Cell Cycle.

[45] Shimizu H., Toma-Fukai S., Kontani K., Katada T., Shimizu T. (2018). GEF mechanism revealed by the structure of SmgGDS-558 and farnesylated RhoA complex and its implication for a chaperone mechanism. Proc. Natl. Acad. Sci. USA.

[46] Shimizu H., Toma-Fukai S., Saijo S., Shimizu N., Kontani K., Katada T., Shimizu T. (2017). Structure-based analysis of the guanine nucleotide exchange factor SmgGDS reveals armadillo-repeat motifs and key regions for activity and GTPase binding. J. Biol. Chem..

[47] Jaiswal M., Dvorsky R., Ahmadian M.R. (2013). Deciphering the molecular and functional basis of Dbl family proteins: A novel systematic approach toward classification of selective activation of the Rho family proteins. J. Biol. Chem..

[48] Barr F., Lambright D.G. (2010). Rab GEFs and GAPs. Curr. Opin. Cell Biol..

[49] Stenmark H. (2009). Rab GTPases as coordinators of vesicle traffic. Nat. Rev. Mol. Cell Biol..

[50] Muller M.P., Goody R.S. (2018). Molecular control of Rab activity by GEFs, GAPs and GDI. Small GTPases.

[51] Pereira-Leal J.B., Seabra M.C. (2000). The mammalian Rab family of small GTPases: Definition of family and subfamily sequence motifs suggests a mechanism for functional specificity in the Ras superfamily. J. Mol. Biol..

[52] Pereira-Leal J.B., Seabra M.C. (2001). Evolution of the Rab family of small GTP-binding proteins. J. Mol. Biol..

[53] Itzen A., Pylypenko O., Goody R.S., Alexandrov K., Rak A. (2006). Nucleotide exchange via local protein unfolding--structure of Rab8 in complex with MSS4. Embo J..

[54] Sato Y., Fukai S., Ishitani R., Nureki O. (2007). Crystal structure of the Sec4p.Sec2p complex in the nucleotide exchanging intermediate state. Proc. Natl. Acad. Sci. USA.

[55] Dong G., Medkova M., Novick P., Reinisch K.M. (2007). A catalytic coiled coil: Structural insights into the activation of the Rab GTPase Sec4p by Sec2p. Mol. Cell.

[56] Rinaldi F.C., Packer M., Collins R. (2015). New insights into the molecular mechanism of the Rab GTPase Sec4p activation. BMC Struct. Biol..

[57] Delprato A., Lambright D.G. (2007). Structural basis for Rab GTPase activation by VPS9 domain exchange factors. Nat. Struct. Mol. Biol..

[58] Cai Y., Chin H.F., Lazarova D., Menon S., Fu C., Cai H., Sclafani A., Rodgers D.W., De La Cruz E.M., Ferro-Novick S. (2008). The structural basis for activation of the Rab Ypt1p by the TRAPP membrane-tethering complexes. Cell.

[59] Gray J.L., von Delft F., Brennan P. (2019). Targeting the Small GTPase Superfamily through their Regulatory Proteins. Angew. Chem. Int. Ed. Engl..

[60] Guo Z., Hou X., Goody R.S., Itzen A. (2013). Intermediates in the guanine nucleotide exchange reaction of Rab8 protein catalyzed by guanine nucleotide exchange factors Rabin8 and GRAB. J. Biol. Chem..

[61] Zhang Z., Zhang T., Wang S., Gong Z., Tang C., Chen J., Ding J. (2014). Molecular mechanism for Rabex-5 GEF activation by Rabaptin-5. Elife.

[62] Goto-Ito S., Morooka N., Yamagata A., Sato Y., Sato K., Fukai S. (2019). Structural basis of guanine nucleotide exchange for Rab11 by SH3BP5. Life Sci. Alliance.

[63] Jenkins M.L., Margaria J.P., Stariha J.T.B., Hoffmann R.M., McPhail J.A., Hamelin D.J., Boulanger M.J., Hirsch E., Burke J.E. (2018). Structural determinants of Rab11 activation by the guanine nucleotide exchange factor SH3BP5. Nat. Commun..

[64] Cherfils J., Menetrey J., Mathieu M., Le Bras G., Robineau S., Beraud-Dufour S., Antonny B., Chardin P. (1998). Structure of the Sec7 domain of the Arf exchange factor ARNO. Nature.

[65] Renault L., Guibert B., Cherfils J. (2003). Structural snapshots of the mechanism and inhibition of a guanine nucleotide exchange factor. Nature.

[66] Weissman J.T., Plutner H., Balch W.E. (2001). The mammalian guanine nucleotide exchange factor mSec12 is essential for activation of the Sar1 GTPase directing endoplasmic reticulum export. Traffic.

[67] McMahon C., Studer S.M., Clendinen C., Dann G.P., Jeffrey P.D., Hughson F.M. (2012). The structure of Sec12 implicates potassium ion coordination in Sar1 activation. J. Biol. Chem..

[68] Renault L., Kuhlmann J., Henkel A., Wittinghofer A. (2001). Structural basis for guanine nucleotide exchange on Ran by the regulator of chromosome condensation (RCC1). Cell.

[69] Nemergut M.E., Mizzen C.A., Stukenberg T., Allis C.D., Macara I.G. (2001). Chromatin docking and exchange activity enhancement of RCC1 by histones H2A and H2B. Science.

[70] Makde R.D., England J.R., Yennawar H.P., Tan S. (2010). Structure of RCC1 chromatin factor bound to the nucleosome core particle. Nature.

[71] Ding L., Lei Y., Han Y., Li Y., Ji X., Liu L. (2016). Vimar Is a Novel Regulator of Mitochondrial Fission through Miro. PLoS Genet..

[72] Margarit S.M., Sondermann H., Hall B.E., Nagar B., Hoelz A., Pirruccello M., Bar-Sagi D., Kuriyan J. (2003). Structural evidence for feedback activation by Ras.GTP of the Ras-specific nucleotide exchange factor SOS. Cell.

[73] Gureasko J., Kuchment O., Makino D.L., Sondermann H., Bar-Sagi D., Kuriyan J. (2010). Role of the histone domain in the autoinhibition and activation of the Ras activator Son of Sevenless. Proc. Natl. Acad. Sci. USA.

[74] Sondermann H., Soisson S.M., Boykevisch S., Yang S.S., Bar-Sagi D., Kuriyan J. (2004). Structural analysis of autoinhibition in the Ras activator Son of sevenless. Cell.

[75] Pacheco-Rodriguez G., Moss J., Vaughan M. (2005). Cytohesin-1: Structure, function, and ARF activation. Methods Enzymol..

[76] DiNitto J.P., Delprato A., Gabe Lee M.T., Cronin T.C., Huang S., Guilherme A., Czech M.P., Lambright D.G. (2007). Structural basis and mechanism of autoregulation in 3-phosphoinositide-dependent Grp1 family Arf GTPase exchange factors. Mol. Cell.

[77] Malaby A.W., van den Berg B., Lambright D.G. (2013). Structural basis for membrane recruitment and allosteric activation of cytohesin family Arf GTPase exchange factors. Proc. Natl. Acad. Sci. USA.

[78] Rossman K.L., Der C.J., Sondek J. (2005). GEF means go: Turning on RHO GTPases with guanine nucleotide-exchange factors. Nat. Rev. Mol. Cell Biol..

[79] Soisson S.M., Nimnual A.S., Uy M., Bar-Sagi D., Kuriyan J. (1998). Crystal structure of the Dbl and pleckstrin homology domains from the human Son of sevenless protein. Cell.

[80] Nimnual A.S., Yatsula B.A., Bar-Sagi D. (1998). Coupling of Ras and Rac guanosine triphosphatases through the Ras exchanger Sos. Science.

[81] Rossman K.L., Worthylake D.K., Snyder J.T., Siderovski D.P., Campbell S.L., Sondek J. (2002). A crystallographic view of interactions between Dbs and Cdc42: PH domain-assisted guanine nucleotide exchange. Embo J..

[82] Derewenda U., Oleksy A., Stevenson A.S., Korczynska J., Dauter Z., Somlyo A.P., Otlewski J., Somlyo A.V., Derewenda Z.S. (2004). The crystal structure of RhoA in complex with the DH/PH fragment of PDZRhoGEF, an activator of the Ca(2+) sensitization pathway in smooth muscle. Structure.

[83] Chhatriwala M.K., Betts L., Worthylake D.K., Sondek J. (2007). The DH and PH domains of Trio coordinately engage Rho GTPases for their efficient activation. J. Mol. Biol..

[84] Baumeister M.A., Rossman K.L., Sondek J., Lemmon M.A. (2006). The Dbs PH domain contributes independently to membrane targeting and regulation of guanine nucleotide-exchange activity. Biochem. J..

[85] Mitin N., Betts L., Yohe M.E., Der C.J., Sondek J., Rossman K.L. (2007). Release of autoinhibition of ASEF by APC leads to CDC42 activation and tumor suppression. Nat. Struct. Mol. Biol..

[86] Murayama K., Shirouzu M., Kawasaki Y., Kato-Murayama M., Hanawa-Suetsugu K., Sakamoto A., Katsura Y., Suenaga A., Toyama M., Terada T. (2007). Crystal structure of the rac activator, Asef, reveals its autoinhibitory mechanism. J. Biol. Chem..

[87] Vigil D., Cherfils J., Rossman K.L., Der C.J. (2010). Ras superfamily GEFs and GAPs: Validated and tractable targets for cancer therapy?. Nat. Rev. Cancer.

[88] Llorca O., Arias-Palomo E., Zugaza J.L., Bustelo X.R. (2005). Global conformational rearrangements during the activation of the GDP/GTP exchange factor Vav3. Embo J..

[89] Yu B., Martins I.R., Li P., Amarasinghe G.K., Umetani J., Fernandez-Zapico M.E., Billadeau D.D., Machius M., Tomchick D.R., Rosen M.K. (2010). Structural and energetic mechanisms of cooperative autoinhibition and activation of Vav1. Cell.

[90] Chrencik J.E., Brooun A., Zhang H., Mathews I.I., Hura G.L., Foster S.A., Perry J.J., Streiff M., Ramage P., Widmer H. (2008). Structural basis of guanine nucleotide exchange mediated by the T-cell essential Vav1. J. Mol. Biol..

[91] Raaijmakers J.H., Bos J.L. (2009). Specificity in Ras and Rap signaling. J. Biol. Chem..

[92] Li P., Martins I.R., Amarasinghe G.K., Rosen M.K. (2008). Internal dynamics control activation and activity of the autoinhibited Vav DH domain. Nat. Struct. Mol. Biol..

[93] Yohe M.E., Rossman K.L., Gardner O.S., Karnoub A.E., Snyder J.T., Gershburg S., Graves L.M., Der C.J., Sondek J. (2007). Auto-inhibition of the Dbl family protein Tim by an N-terminal helical motif. J. Biol. Chem..

[94] Yohe M.E., Rossman K., Sondek J. (2008). Role of the C-terminal SH3 domain and N-terminal tyrosine phosphorylation in regulation of Tim and related Dbl-family proteins. Biochemistry.

[95] Jackson L.P., Kelly B.T., McCoy A.J., Gaffry T., James L.C., Collins B.M., Honing S., Evans P.R., Owen D.J. (2010). A large-scale conformational change couples membrane recruitment to cargo binding in the AP2 clathrin adaptor complex. Cell.

[96] Yamaguchi K., Imai K., Akamatsu A., Mihashi M., Hayashi N., Shimamoto K., Kawasaki T. (2012). SWAP70 functions as a Rac/Rop guanine nucleotide-exchange factor in rice. Plant J..

[97] Gu Y., Li S., Lord E.M., Yang Z. (2006). Members of a novel class of Arabidopsis Rho guanine nucleotide exchange factors control Rho GTPase-dependent polar growth. Plant. Cell.

[98] Hanawa-Suetsugu K., Kukimoto-Niino M., Mishima-Tsumagari C., Akasaka R., Ohsawa N., Sekine S., Ito T., Tochio N., Koshiba S., Kigawa T. (2012). Structural basis for mutual relief of the Rac guanine nucleotide exchange factor DOCK2 and its partner ELMO1 from their autoinhibited forms. Proc. Natl. Acad. Sci. USA.

[99] Lu M., Kinchen J.M., Rossman K.L., Grimsley C., deBakker C., Brugnera E., Tosello-Trampont A.C., Haney L.B., Klingele D., Sondek J. (2004). PH domain of ELMO functions in trans to regulate Rac activation via Dock180. Nat. Struct. Mol. Biol..

[100] Lu M., Kinchen J.M., Rossman K.L., Grimsley C., Hall M., Sondek J., Hengartner M.O., Yajnik V., Ravichandran K.S. (2005). A Steric-inhibition model for regulation of nucleotide exchange via the Dock180 family of GEFs. Curr. Biol..

[101] Medina F., Carter A.M., Dada O., Gutowski S., Hadas J., Chen Z., Sternweis P.C. (2013). Activated RhoA is a positive feedback regulator of the Lbc family of Rho guanine nucleotide exchange factor proteins. J. Biol. Chem..

[102] Chen Z., Medina F., Liu M.Y., Thomas C., Sprang S.R., Sternweis P.C. (2010). Activated RhoA binds to the pleckstrin homology (PH) domain of PDZ-RhoGEF, a potential site for autoregulation. J. Biol. Chem..

[103] Dada O., Gutowski S., Brautigam C.A., Chen Z., Sternweis P.C. (2018). Direct regulation of p190RhoGEF by activated Rho and Rac GTPases. J. Struct. Biol..

[104] Lin Q., Yang W., Baird D., Feng Q., Cerione R.A. (2006). Identification of a DOCK180-related guanine nucleotide exchange factor that is capable of mediating a positive feedback activation of Cdc42. J. Biol. Chem..

[105] De Rooij J., Zwartkruis F.J., Verheijen M.H., Cool R.H., Nijman S.M., Wittinghofer A., Bos J.L. (1998). Epac is a Rap1 guanine-nucleotide-exchange factor directly activated by cyclic AMP. Nature.

[106] Kawasaki H., Springett G.M., Mochizuki N., Toki S., Nakaya M., Matsuda M., Housman D.E., Graybiel A.M. (1998). A family of cAMP-binding proteins that directly activate Rap1. Science.

[107] Burton J.L., Burns M.E., Gatti E., Augustine G.J., De Camilli P. (1994). Specific interactions of Mss4 with members of the Rab GTPase subfamily. Embo J..

[108] Coppola T., Perret-Menoud V., Gattesco S., Magnin S., Pombo I., Blank U., Regazzi R. (2002). The death domain of Rab3 guanine nucleotide exchange protein in GDP/GTP exchange activity in living cells. Biochem. J..

[109] Muller-Pillasch F., Zimmerhackl F., Lacher U., Schultz N., Hameister H., Varga G., Friess H., Buchler M., Adler G., Gress T.M. (1997). Cloning of novel transcripts of the human guanine-nucleotide-exchange factor Mss4: In situ chromosomal mapping and expression in pancreatic cancer. Genomics.

[110] Wada M., Nakanishi H., Satoh A., Hirano H., Obaishi H., Matsuura Y., Takai Y. (1997). Isolation and characterization of a GDP/GTP exchange protein specific for the Rab3 subfamily small G proteins. J. Biol. Chem..

[111] Walch-Solimena C., Collins R.N., Novick P.J. (1997). Sec2p mediates nucleotide exchange on Sec4p and is involved in polarized delivery of post-Golgi vesicles. J. Cell Biol..

[112] Esters H., Alexandrov K., Iakovenko A., Ivanova T., Thoma N., Rybin V., Zerial M., Scheidig A.J., Goody R.S. (2001). Vps9, Rabex-5 and DSS4: Proteins with weak but distinct nucleotide-exchange activities for Rab proteins. J. Mol. Biol..

[113] Burton J., Roberts D., Montaldi M., Novick P., De Camilli P. (1993). A mammalian guanine-nucleotide-releasing protein enhances function of yeast secretory protein Sec4. Nature.

[114] Moya M., Roberts D., Novick P. (1993). DSS4-1 is a dominant suppressor of sec4-8 that encodes a nucleotide exchange protein that aids Sec4p function. Nature.

[115] Nuoffer C., Wu S.K., Dascher C., Balch W.E. (1997). Mss4 does not function as an exchange factor for Rab in endoplasmic reticulum to Golgi transport. Mol. Biol. Cell.

[116] Gulbranson D.R., Davis E.M., Demmitt B.A., Ouyang Y., Ye Y., Yu H., Shen J. (2017). RABIF/MSS4 is a Rab-stabilizing holdase chaperone required for GLUT4 exocytosis. Proc. Natl. Acad. Sci. USA.

[117] Yu H., Schreiber S.L. (1995). Structure of guanine-nucleotide-exchange factor human Mss4 and identification of its Rab-interacting surface. Nature.

[118] Zhu Z., Dumas J.J., Lietzke S.E., Lambright D.G. (2001). A helical turn motif in Mss4 is a critical determinant of Rab binding and nucleotide release. Biochemistry.

[119] Williams C.L. (2003). The polybasic region of Ras and Rho family small GTPases: A regulator of protein interactions and membrane association and a site of nuclear localization signal sequences. Cell Signal..

[120] Ogita Y., Egami S., Ebihara A., Ueda N., Katada T., Kontani K. (2015). Di-Ras2 Protein Forms a Complex with SmgGDS Protein in Brain Cytosol in Order to Be in a Low Affinity State for Guanine Nucleotides. J. Biol. Chem..

[121] Bergom C., Hauser A.D., Rymaszewski A., Gonyo P., Prokop J.W., Jennings B.C., Lawton A.J., Frei A., Lorimer E.L., Aguilera-Barrantes I. (2016). The Tumor-suppressive Small GTPase DiRas1 Binds the Noncanonical Guanine Nucleotide Exchange Factor SmgGDS and Antagonizes SmgGDS Interactions with Oncogenic Small GTPases. J. Biol. Chem..

[122] Tew G.W., Lorimer E.L., Berg T.J., Zhi H., Li R., Williams C.L. (2008). SmgGDS regulates cell proliferation, migration, and NF-kappaB transcriptional activity in non-small cell lung carcinoma. J. Biol. Chem..

[123] Schuld N.J., Hauser A.D., Gastonguay A.J., Wilson J.M., Lorimer E.L., Williams C.L. (2014). SmgGDS-558 regulates the cell cycle in pancreatic, non-small cell lung, and breast cancers. Cell Cycle.

[124] Zhi H., Yang X.J., Kuhnmuench J., Berg T., Thill R., Yang H., See W.A., Becker C.G., Williams C.L., Li R. (2009). SmgGDS is up-regulated in prostate carcinoma and promotes tumour phenotypes in prostate cancer cells. J. Pathol..

[125] Hauser A.D., Bergom C., Schuld N.J., Chen X., Lorimer E.L., Huang J., Mackinnon A.C., Williams C.L. (2014). The SmgGDS splice variant SmgGDS-558 is a key promoter of tumor growth and RhoA signaling in breast cancer. Mol. Cancer Res..

[126] Peifer M., Berg S., Reynolds A.B. (1994). A repeating amino acid motif shared by proteins with diverse cellular roles. Cell.

[127] Kuhlmann N., Wroblowski S., Knyphausen P., de Boor S., Brenig J., Zienert A.Y., Meyer-Teschendorf K., Praefcke G.J., Nolte H., Krüger M. (2016). Structural and mechanistic insights into the regulation of the fundamental Rho regulator RhoGDIα by lysine acetylation. J. Biol. Chem..

[128] Garcia-Mata R., Boulter E., Burridge K. (2011). The ‘invisible hand’: Regulation of RHO GTPases by RHOGDIs. Nat. Rev. Mol. Cell Biol..

[129] Dharmaiah S., Bindu L., Tran T.H., Gillette W.K., Frank P.H., Ghirlando R., Nissley D.V., Esposito D., McCormick F., Stephen A.G. (2016). Structural basis of recognition of farnesylated and methylated KRAS4b by PDEδ. Proc. Natl. Acad. Sci. USA.

[130] Wang M., Casey P.J. (2016). Protein prenylation: Unique fats make their mark on biology. Nat. Rev. Mol. Cell Biol..

[131] Ahearn I.M., Haigis K., Bar-Sagi D., Philips M.R. (2012). Regulating the regulator: Post-translational modification of RAS. Nat. Rev. Mol. Cell Biol..

